# Highly efficient CRISPR systems for loss-of-function and gain-of-function research in pear calli

**DOI:** 10.1093/hr/uhac148

**Published:** 2022-06-30

**Authors:** Meiling Ming, Hongjun Long, Zhicheng Ye, Changtian Pan, Jiali Chen, Rong Tian, Congrui Sun, Yongsong Xue, Yingxiao Zhang, Jiaming Li, Yiping Qi, Jun Wu

**Affiliations:** College of Horticulture, State Key Laboratory of Crop Genetics and Germplasm Enhancement, Nanjing Agricultural University, Nanjing 210095, China; College of Horticulture, State Key Laboratory of Crop Genetics and Germplasm Enhancement, Nanjing Agricultural University, Nanjing 210095, China; College of Horticulture, State Key Laboratory of Crop Genetics and Germplasm Enhancement, Nanjing Agricultural University, Nanjing 210095, China; Department of Plant Science and Landscape Architecture, University of Maryland, College Park, MD 20742, USA; College of Horticulture, State Key Laboratory of Crop Genetics and Germplasm Enhancement, Nanjing Agricultural University, Nanjing 210095, China; College of Horticulture, State Key Laboratory of Crop Genetics and Germplasm Enhancement, Nanjing Agricultural University, Nanjing 210095, China; College of Horticulture, State Key Laboratory of Crop Genetics and Germplasm Enhancement, Nanjing Agricultural University, Nanjing 210095, China; College of Horticulture, State Key Laboratory of Crop Genetics and Germplasm Enhancement, Nanjing Agricultural University, Nanjing 210095, China; Department of Plant Science and Landscape Architecture, University of Maryland, College Park, MD 20742, USA; College of Horticulture, State Key Laboratory of Crop Genetics and Germplasm Enhancement, Nanjing Agricultural University, Nanjing 210095, China; Department of Plant Science and Landscape Architecture, University of Maryland, College Park, MD 20742, USA; Institute for Bioscience and Biotechnology Research, University of Maryland, Rockville, MD 20850, USA; College of Horticulture, State Key Laboratory of Crop Genetics and Germplasm Enhancement, Nanjing Agricultural University, Nanjing 210095, China

## Abstract

CRISPR/Cas systems have been widely used for genome engineering in many plant species. However, their potentials have remained largely untapped in fruit crops, particularly in pear, due to the high levels of genomic heterozygosity and difficulties in tissue culture and stable transformation. To date, only a few reports on the application of the CRISPR/Cas9 system in pear have been documented, and have shown very low editing efficiency. Here we report a highly efficient CRISPR toolbox for loss-of-function and gain-of-function research in pear. We compared four different CRISPR/Cas9 expression systems for loss-of-function analysis and identified a potent system that showed nearly 100% editing efficiency for multi-site mutagenesis. To expand the targeting scope, we further tested different CRISPR/Cas12a and Cas12b systems in pear for the first time, albeit with low editing efficiency. In addition, we established a CRISPR activation (CRISPRa) system for multiplexed gene activation in pear calli for gain-of-function analysis. Furthermore, we successfully engineered the anthocyanin and lignin biosynthesis pathways using both CRISPR/Cas9 and CRISPRa systems in pear calli. Taking these results together, we have built a highly efficient CRISPR toolbox for genome editing and gene regulation, paving the way for functional genomics studies as well as molecular breeding in pear.

## Introduction

CRISPR (clustered regularly interspaced short palindromic repeats)/Cas9 (CRISPR-associated protein), a Class 2 type II CRISPR system, can be easily programmed to introduce DNA double-strand breaking (DSB) at the desired target site [1]. CRISPR/Cas9-mediated genome editing has been widely adopted across diverse species due to its high efficiency, specificity, simplicity, and versatility of multiplexing [2–5]. In plants, CRISPR/Cas9-mediated genome editing has greatly advanced functional genomics and crop improvement [5–7]. For example, CRISPR/Cas9 systems make it possible to shorten breeding cycles and accelerate plant domestication [8, 9], increase crop yield and improve food quality [10, 11], enhance resistance to biotic and abiotic stresses [12, 13], engineer metabolic pathways [14, 15], and break self-incompatibility [16].

To achieve the full potential of CRISPR/Cas systems in plants, intense efforts have been made to improve editing efficiencies by optimizing expression cassettes and regulatory elements. Previous studies have demonstrated high activity of plant codon-optimized Cas9 driven by Pol II (RNA polymerase II) promoters, including cauliflower mosaic virus (CaMV) 35S, ubiquitin, *YAO*, andthe egg-cell specific promoter [17–19]. Single guide RNA(sgRNA) expression is usually driven by a Pol III promotersuch as U3 or U6 [17–19]. However, these promoters have been demonstrated to be species-dependent for the efficiencies of CRISPR/Cas systems [20, 21].

While the canonical CRISPR/Cas9 system recognizes an NGG protospacer adjacent motif (PAM) [22], Cas12 systems such as Cas12a (a Class 2 type V-A CRISPRsystem) and Cas12b (a Class 2 type V-B CRISPR system) prefer T-rich PAMs [23, 24]. As complements of Cas9, CRISPR/Cas12a and Cas12b systems have shown comparable activity and higher targeting specificity, makingit possible to generate staggered DSBs and larger deletions in many plant species [3, 24]. Moreover, Cas12a is more suitable for multiplexed genome editing, since the CRISPR RNA (crRNA) array is short in length and can beprocessed by its RNase activity. LbCas12a and AaCas12b have been shown to be most reliable and efficient forgenome editing in plants [24–27]. Recently, a new Cas12a ortholog, Mb2Cas12a, has been reported in rice, showing relaxed PAM requirements, high editing activity andtolerance of relatively low temperatures [28]. However, neither CRISPR/Cas12a nor Cas12b systems have been demonstrated in pear.

Although CRISPR/Cas systems have been commonly used to generate loss-of-function mutations, they have also been repurposed as a programmable platform for gain-of-function analysis by transcriptional activation of target genes. The conventional gene overexpression approach by expressing the target gene with a constitutive promoter is laborious and challenging for multigene upregulation. CRISPRa systems, based on a deactivated Cas (dCas) protein fused with transcriptional activators, have shown efficient activation activity in several model plants [29–34]. dCas protein has lost its ability to cut DNA due to mutations in the nuclease domain, but it remains competent for RNA-guided DNA binding without DSB in the target gene. The target gene is then activated by transcriptional activators without sequence mutation. Importantly, CRISPRa systems allow simultaneous gene activation by assembling multiple sgRNAs targeting promoter regions of target genes. Recently, CRISPR-Act3.0, a third-generation CRISPRa system, showed potent single or multiplexed gene activation in rice, *Arabidopsis*, and tomato [35], further broadening the application of CRISPRa in plants.

Pear is an economically important fruit crop that belongs to the genus *Pyrus* in the Rosaceae family and has been widely cultivated in the world for >3000 years [36]. Genome editing holds great promise for revolutionizing pear genetics and breeding. Yet only a few studies have reported CRISPR/Cas9-mediated genome editing in pear and the editing efficiency remains very low [37]. In this study we compared four different CRISPR/Cas9 expression systems and identified a potent system that showed nearly 100% editing efficiency even in a multiplexed setting using a stable transgenic pear callus system. Moreover, we benchmarked this efficient CRISPR/Cas9 system with comparison to different CRISPR/Cas12a and Cas12b systems in pear calli. As a demonstration, we successfully engineered anthocyanin and lignin biosynthesis by knocking out several key pathway genes in pear calli. To establish an effective gain-of-function system, we successfully applied the CRISPR-Act3.0 system to activate genes involved in anthocyanin biosynthesis and observed phenotypic changes in pear calli. To our knowledge, this is the first demonstration of a CRISPRa system in pear. In summary, this study successfully established efficient CRISPR systems for gene loss-of-function and gain-of-function studies in pear.

## Results

### Comprehensive analysis of four different CRISPR/Cas9 expression systems for genome editing in pear

**Figure 1:**
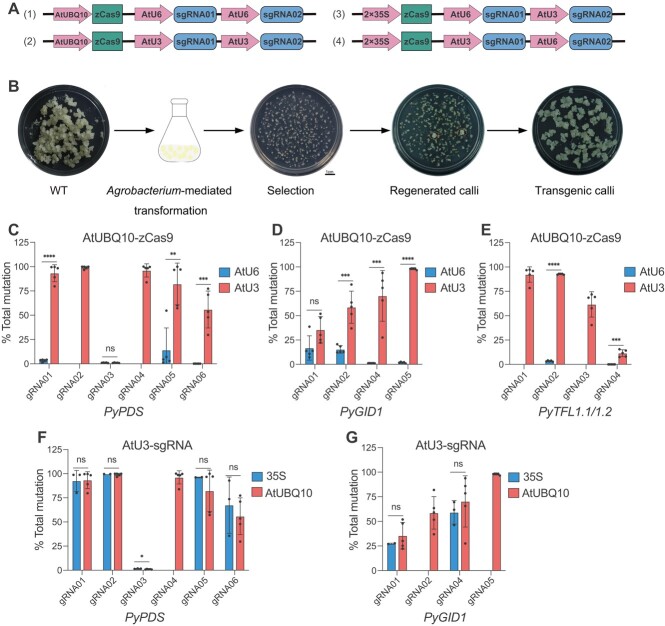
Comparison of different CRISPR/Cas9 systems for genome editing in pear calli. (A) Schematic illustration of different CRISPR/Cas9 systems with different promoters for Cas9 and sgRNA expression. AtUBQ10, *Arabidopsis* ubiquitin promoter; 35S, CAMV 35S promoter; AtU3, *Arabidopsis* U3 promoter; AtU6, *Arabidopsis* U6 promoter; zCas9, maize codon-optimized SpCas9. (B) Stable transformation of pear calli. WT, wild-type pear calli. A mass of regenerated calli highlighted by the orange dotted circle is considered to be an independent stable line. (C–E) Comparison of mutation frequencies of AtU3- and AtU6-based sgRNA expression for CRISPR/Cas9 systems in pear calli. zCas9 is driven by AtUBQ10 promoter. A total of six, four, and four unique sgRNAs were designed for *PyPDS* (C)*, PyGID1* (D), and *PyTFL1.1*/*1.2* (E), respectively. (F, G) Comparison of mutation frequencies of AtUBQ10- and 35S-based Cas9 expression for CRISPR/Cas9 systems in pear calli. The sgRNAs are driven by AtU3 promoter. A total of six and four unique sgRNAs were designed for *PyPDS* (F) and *PyGID1* (G), respectively. All data are derived from high-throughput amplicon deep sequencing and presented as mean ± standard deviation (*n* = 3–5 independent lines). ^ns^*P* > .05, ^*^P < .05, ^**^P < .01, ^***^P < .001, ^****^P < .0001; two-tailed Student’s *t*-test.

To improve CRISPR/Cas9-mediated genome editing in pear, we constructed four different CRISPR/Cas9 expression systems (Fig. 1A), which allowed us to compare two Pol II promoters (35S and AtUBQ10) for Cas9 expression and two Pol III promoters (AtU3 and AtU6) for sgRNA expression. Four genes, *PyPDS*, *PyGID1*, *PyTFL1.1*, and *PyTFL1.2* were chosen as target genes. Loss-of-function of *PDS* (phytoene desaturase), *GID1* (gibberellic acid receptor), and *TFL1* (terminal flower 1) would produce albino [37], dwarfing [38], and early-flowering [37] phenotypes, respectively. The *PyPDS*, *PyGID1*, and *PyTFL1.1/1.2* DNA sequences of pear callus showed some single-nucleotide polymorphisms (SNPs) compared with the reference genome (Supplementary Data Figs S1, S2, and S3). The DNA regions without SNPs were considered for targeted mutagenesis. We designed six and four sgRNAs targeting *PyPDS* and *PyGID1* exons, respectively (Supplementary Data Fig. S4A). Considering the high sequence identity between *PyTFL1.1* and *PyTFL1.2* (Supplementary Data Fig. S3), two sgRNAs (sgRNA01 and sgRNA02) were designed to target the common region of *PyTFL1.1* and *PyTFL1.2*, and one sgRNA (sgRNA03 or sgRNA04) was designed to specifically target *PyTFL1.1* or *PyTFL1.2* (Supplementary Data Fig. S4A). A total of 26 CRISPR/Cas9 T-DNA vectors were constructed to compare editing efficiencies using different Pol II and Pol III promoters in pear. In each T-DNA vector, two sgRNAs were simultaneously expressed to target each gene (Supplementary Data Fig. S4B).

We first tried *Agrobacterium*-mediated stable transformation of pear plants. A total of 2860 leaves were used for co-culture, and 1130 regenerated seedlings were harvested. However, no transgenic plant was identified by screening of 1130 regenerated seedlings with 3 mg/L hygromycin B (all seedlings died; data not shown), resulting from the extremely low transformation efficiency.

We next assessed these four different CRISPR/Cas9 expression systems for genome editing in stable dedifferentiated pear calli (Fig. 1B). The CRISPR/Cas9 components in the stable transgenic calli were detected using PCR with vector-specific primers (Supplementary Data Fig. S5). The resistance of T-DNA vectors pLR01–16 with Cas9 driven by the AtUBQ10 promoter in plants is to hygromycin, and that of pLR17–26 with Cas9 driven by 35S promoter is to kanamycin. However, we found that 150 mg/L kanamycin had no selection pressure compared with 15 mg/L hygromycin B for obtaining transgenic pear calli. We subsequently replaced the kanamycin resistance gene in T-DNA vectors pLR17–26 with the hygromycin resistance gene and renamed them to pLR55–64 (Supplementary Data Fig. S6B). During callus regeneration, a mass of regenerated calli, highlighted by an orange dotted circle in Fig. 1B, is considered to be an independent stable line. An independent stable line always contains more than one single cell. Stable transgenic callus lines were obtained for nearly all T-DNA vectors (Supplementary Data Figs S5 and S6A). Mutation frequencies at the *PyPDS*-gRNA05 site were first detected by PCR–restriction fragment length polymorphism (PCR–RFLP) and Sanger sequencing analysis (Supplementary Data Fig. S7). PCR–RFLP analysis showed that the vector pLR06 with the AtU3 promoter exhibited 100 and 50% editing efficiency in two randomly tested independent lines. By contrast, the vector pLR05 with the AtU6 promoter exhibited no editing in two selected lines (Supplementary Data Fig. S7A). Sanger sequencing analysis further confirmed the results of PCR–RFLP analysis (Supplementary Data Fig. S7B).

To further assess the editing efficiencies of these four different CRISPR/Cas9 expression systems, we determined the mutation frequencies at all target sites in stable pear calli by high-throughput amplicon deep sequencing. Three to five independent lines were genotyped at each target site. In AtUBQ10-zCas9-mediated pear calli, mutation frequencies at five out of six sites of *PyPDS* were extremely high (nearly 100%) when sgRNAs were driven by the AtU3 promoter (Fig. 1C). By contrast, mutation frequencies at the same sites of *PyPDS* were extremely low (nearly 0%) when sgRNAs were driven by the AtU6 promoter (Fig. 1C). Consistently, mutation analysis of the other three target genes, *PyGID1* and *PyTFL1.1/1.2*, also showed that the AtU3 promoter induced significantly higher editing efficiencies than the AtU6 promoter (Fig. 1D and E). These results suggest that the AtU3 promoter outperforms the AtU6 promoter for targeted mutagenesis in pear calli. Then we compared the efficiency of 35S- and AtUBQ10-based Cas9 expression with the AtU3-sgRNA module. At the five *PyPDS* sites and two *PyGID1* sites, the 35S and AtUBQ10 promoters induced comparable editing frequencies (Fig. 1F and G). Impressively, mutation frequencies at 8 out of 14 test sites were nearly 100% when AtU3 was used for sgRNA expression and AtUBQ10 was used for Cas9 expression (Fig. 1C–E). Taking these results together, the combination of 35S- or AtUBQ10-Cas9 and AtU3-sgRNA modules represents a potent CRISPR/Cas9 system for loss-of-function analysis in pear.

### Mutation types of potent CRISPR/Cas9 systems

**Figure 2:**
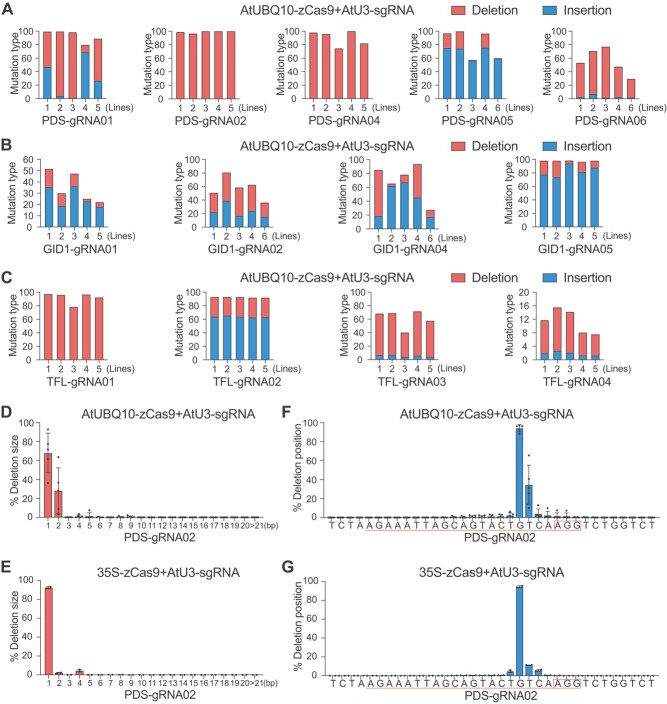
Mutation types of different CRISPR/Cas9 systems for genome editing in pear calli. (A–C) Deletion and insertion frequencies of the CRISPR/Cas9 system with the AtUBQ10 and AtU3 promoters. A total of five, four and four target sites were analyzed for *PyPDS* (A), *PyGID1* (B), and *PyTFL1.1*/*1.2* (C), respectively. (D, E) Comparison of deletion sizes of AtUBQ10- and 35S-based zCas9 at the *PyPDS-*gRNA02 site. The sgRNA is driven by the AtU3 promoter. (F, G) Comparison of deletion position of AtUBQ10- and 35S-based zCas9 at the *PyPDS-*gRNA02 site. The sgRNA is driven by the AtU3 promoter. PAM and protospacer sequences are circled and underlined, respectively. All data are derived from high-throughput amplicon deep sequencing and presented as the mean ± standard deviation (*n* = 3–5 independent lines).

We assessed the mutation types of the two potent CRISPR/Cas9 systems, AtUBQ10-zCas9 and 35S-zCas9, coupled with AtU3-sgRNA. High-throughput sequencing results showed that both CRISPR/Cas9 systems mainly induced insertion and deletion mutations, which varied across the target sites (Fig. 2A–C, Supplementary Data Fig. S8). For each target site, the occurrence and frequency of insertions and deletions were relatively consistent across five independent lines (Fig. 2A–C), suggesting protospacer sequences largely dictate the editing outcomes. Occasionally, there is a slight difference observed between the AtUBQ10 and 35S promoters. For example, at the *PyPDS*-gRNA02 site Cas9 driven by AtUBQ10 mainly generated 1- or 2-bp deletions, while Cas9 driven by 35S mainly generated 1-bp deletions (Fig. 2D and E). Such a difference might be attributed to discrepant activity of Cas9 in these two constructs. At this site, the most frequent deletion position is the fourth base upstream of the PAM site (Fig. 2F and G) for both systems, one base pair upstream of the DSB site.

### CRISPR/Cas9 is far superior to Cas12a and Cas12b for genome editing in pear calli

**Figure 3:**
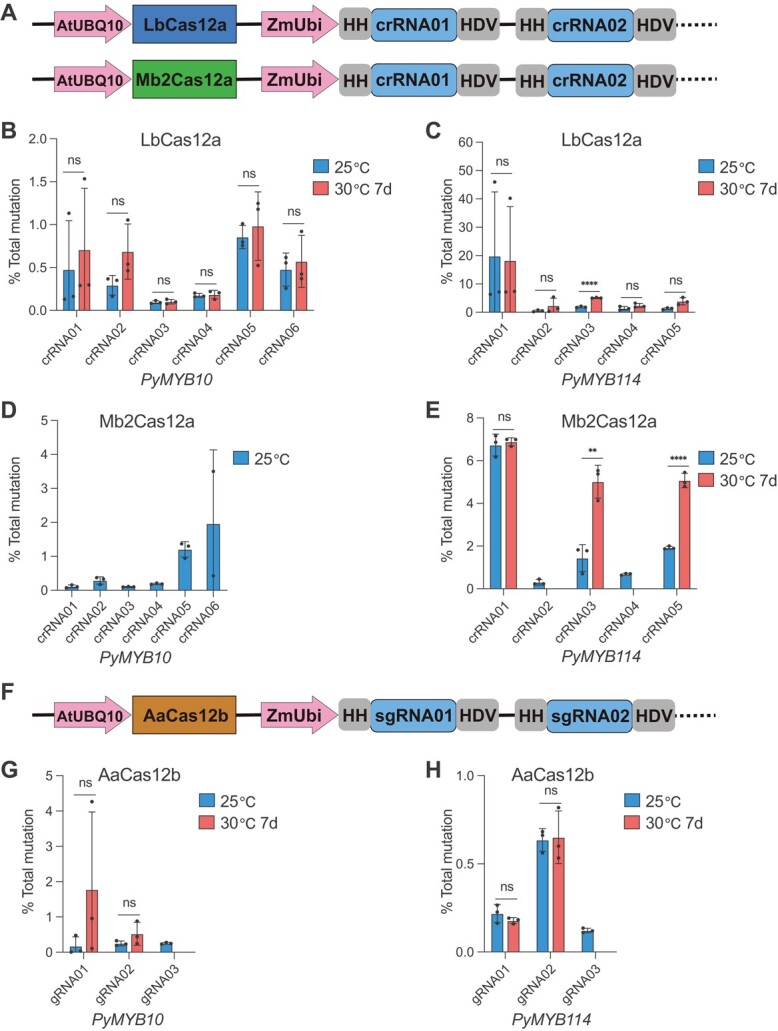
Analysis of CRISPR/Cas12a and Cas12b systems for genome editing in pear calli. (A) Schematic illustration of the dual Pol II promoter system for expression of Cas12a and crRNAs. AtUBQ10, *Arabidopsis* ubiquitin promoter; ZmUbi, maize ubiquitin promoter; HH, hammerhead ribozyme; HDV, hepatitis delta virus ribozyme. (B–E) Analysis of mutation frequencies of LbCas12a (B, C) and Mb2Cas12a (D, E) at 25 and 30°C in stable pear calli. Six and five different crRNAs were designed for *PyMYB10* (B, D) and *PyMYB114* (C, E), respectively. (F) Schematic illustration of the dual Pol II promoter system for expression of Cas12b and sgRNAs. (G, H) Analysis of mutation frequencies of AaCas12b at 25 and 30°C in stable pear calli. Three different sgRNAs were designed for *PyMYB10* (G) and *PyMYB114* (H). All data are derived from high-throughput amplicon deep sequencing and presented as the mean ± standard deviation (*n* = 3 independent lines). ^ns^*P* > .05, ^*^P < .05, ^**^P < .01, ^***^P < .001, ^****^P < .0001; two-tailed Student’s *t*-test.

To test CRISPR/Cas12a and Cas12b systems in pear, we focused on LbCas12a, Mb2Cas12a, and AaCas12b, which showed high editing efficiencies in plants [24, 25, 28]. We expressed the rice codon-optimized LbCas12a, Mb2Cas12a, and AaCas12b with the AtUBQ10 promoter. The crRNA (for Cas12a) or sgRNA (for Cas12b) array containing multiple crRNAs or sgRNAs flanked by hammerhead (HH) and hepatitis delta virus (HDV) ribozymes was expressed under a maize ubiquitin promoter (ZmUbi) (Fig. 3A and F). We targeted exons of anthocyanin-regulating genes, *PyMYB10* and *PyMYB114* [39], and avoided all SNPs (Supplementary Data Fig. S9). For Cas12a, we designed six and five 23-nucleotide (nt) crRNAs with TTTV PAMs for *PyMYB10* and *PyMYB114*, respectively. For Cas12b, we designed three 20-nt sgRNAs with VTTV PAMs for both *PyMYB10* and *PyMYB114* (Supplementary Data Fig. S10A). Two or four crRNAs for Cas12a or two sgRNAs for Cas12b were simultaneously expressed in each T-DNA vector for single or multiplexed gene mutation (Supplementary Data Fig. S10B). The CRISPR/Cas12a and Cas12b components in the stable transgenic calli were first confirmed using PCR with vector-specific primers (Supplementary Data Fig. S11). In these transgenic calli, the editing efficiencies were relatively low for LbCas12a, Mb2Cas12a, and AaCas12b (Fig. 3B–E, G and H). To investigate whether higher temperature could improve the efficiency of LbCas12a, Mb2Cas12a, and AaCas12b, the stable pear calli cultured at 25°C were then incubated at 30°C. After 7 days of incubation, the editing efficiencies at all target sites remained at very low levels, although several of them were enhanced (Fig. 3B–E, G and H). These data demonstrated that, compared with CRISPR/Cas9, the current CRISPR/Cas12a and Cas12b systems need to be optimized for genome editing in pear. With such a comparison we have benchmarked our highly efficient CRISPR/Cas9 system as the preferred genome editing system in pear.

### Highly efficient multiplexed editing of anthocyanin and lignin biosynthetic genes in pear calli

**Figure 4:**
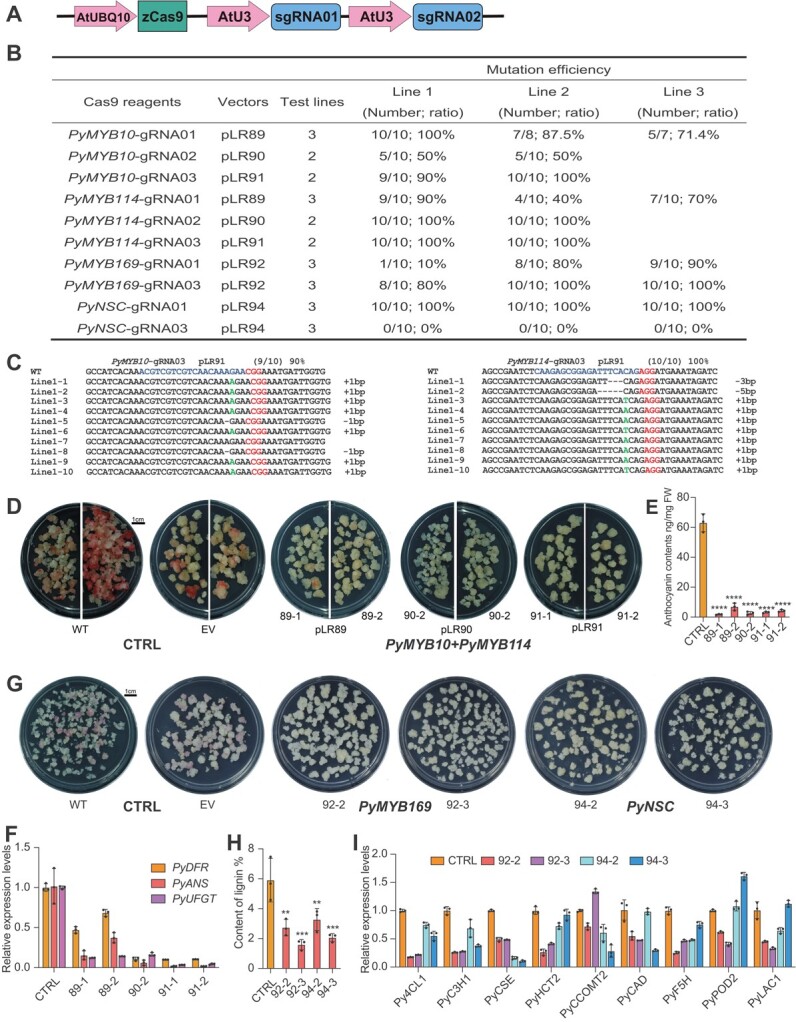
Multiplexed anthocyanin and lignin biosynthetic gene knockout by CRISPR/Cas9 system in pear calli. (A) Schematic illustration of CRISPR/Cas9 system for multiplexed gene or site knockout in pear calli. (B) Summary of mutation results of the CRISPR/Cas9 system at different sites in stable pear calli. Mutation efficiencies were generated using Sanger sequencing. The number represents mutated clones/sequenced clones. (C) Mutation type and frequency of each mutation at the *PyMYB10*-gRNA03 and *PyMYB114*-gRNA03 sites. The PAM sequence is highlighted in red and the target sequence in blue. WT, wild-type sequence. Dash indicates a 1-bp deletion; green DNA bases indicate insertion. (D) Phenotypes of *PyMYB10* and *PyMYB114* knockout calli with continuous light treatment for 7 days. Treatment medium was MS solid medium without nitrogen but containing 200 μM/L MeJA. (E) Anthocyanin contents of CRISPR/Cas9-mediated and CTRL pear calli. (F) Relative transcript level of anthocyanin biosynthetic genes in CRISPR/Cas9-mediated and CTRL pear calli. (G) Phenotypes of *PyMYB169* and *PyNSC* knockout calli with continuous dark treatment for 20 days. (H) Lignin contents in CRISPR/Cas9-mediated and CTRL pear calli. (I) Relative transcript level of lignin biosynthetic genes in CRISPR/Cas9-mediated and CTRL calli. WT calli and calli harboring T-DNA vector without sgRNA (EV) were used as controls (CTRL). *PyGAPDH* was used as the endogenous control gene. All data are presented as the mean ± standard deviation (*n* = 3 independent experiments). ^ns^*P* > .05, ^*^P < .05, ^**^P < .01, ^***^P < .001, ^****^P < .0001, two-tailed Student’s *t*-test.

A callus system can accelerate the process of gene functional study because it is a homologous system amenable to genetic engineering [40–43]. We reasoned that application of our highly efficient CRISPR/Cas9 system in stable pear calli represents an effective genetic method for gene functional studies in pear. To demonstrate such an application, we constructed five CRISPR/Cas9 vectors targeting *PyMYB10*, *PyMYB114*, *PyMYB169*, and *PyNSC* (Fig. 4A). *PyMYB10* and *PyMYB114* are key transcription factors that are involved in enhancing anthocyanin biosynthesis, while *PyMYB169* and *PyNSC* are important transcription factors involved in enhancing lignin biosynthesis [39, 44, 45]. Analysis of *PyMYB10*, *PyMYB114*, *PyMYB169*, and *PyNSC* DNA sequences in pear calli showed that there are some SNPs compared with the reference genome (Supplementary Data Figs S9 and S12). We designed three or two sgRNAs targeting exons without SNPs (except for *PyNSC-*gRNA03) for each target gene (Supplementary Data Fig. S13A). Note that there is a 1-bp mismatched nucleotide in *PyNSC-*gRNA03, which was intentionally designed to assess targeting specificity (Supplementary Data Figs S12B and S13A). Two sgRNAs were simultaneously expressed in each T-DNA vector for single or multiplexed gene mutation (Supplementary Data Fig. S13B). The T-DNA vectors pLR89, pLR90, and pLR91 were designed to target *PyMYB10* and *PyMYB114* simultaneously. The T-DNA vectors pLR92 and pLR94 were designed to target *PyMYB169* and *PyNSC*, respectively (Supplementary Data Fig. S13B). The CRISPR/Cas9 components in the stable pear calli were detected using PCR with vector-specific primers (Supplementary Data Fig. S14). Two or three independent transgenic lines were genotyped at each target site by Sanger sequencing. Overall, mutation frequencies were very high (80–100%) at all target sites except the *PyMYB10-*gRNA02 site, with 50% editing efficiency (Fig. 4B). For *PyNSC-*gRNA03 with 1-bp mismatched nucleotide, none of the three transgenic callus lines showed detectable editing (Fig. 4B, Supplementary Data Fig. S16), suggesting high specificity of the CRISPR/Cas9 system. Impressively, mutation frequencies at six out of nine sites were up to 100% (Fig. 4B). Further mutation analysis revealed that 1-bp insertions and deletions have occurred predominantly at most target sites (Fig. 4C, Supplementary Data Figs S15 and S16). Some large deletions (>20 bp) were also identified at some target sites by Sanger sequencing (Supplementary Data Figs S15 and S16).

Such high-frequency targeted mutagenesis suggests that we can easily generate loss-of-function callus mutants for genetic studies. To this end, we selected the knockout callus lines of *PyMYB10* and *PyMYB114* for phenotypic characterization. The wild-type (WT) calli and calli harboring T-DNA vector without sgRNA (EV) were used as controls. The pear calli were grown on treatment medium under a continuous light condition at 17°C. The treatment medium was Murashige and Skoog (MS) solid medium without nitrogen but containing 200 μM methyl jasmonate (MeJA). After 7 days of treatment, the control pear calli appeared red, indicating strong accumulation of anthocyanin, whereas the calli with CRISPR/Cas9-mediated *PyMYB10* and *PyMYB114* knocked out showed no or weak accumulation of anthocyanin (Fig. 4D and E). Consequently, the expression levels of anthocyanin biosynthesis-related enzyme-encoding genes *PyDFR*, *PyANS*, and *PyUFGT* were substantially lower in the *PyMYB10* and *PyMYB114* knockout calli than in controls (Fig. 4F), suggesting these genes are regulated by *PyMYB10* and *PyMYB114* [39].

We also identified the phenotypes of CRISPR/Cas9-mediated *PyMYB169* or *PyNSC* knockout calli. The WT calli and EV calli were used as controls. The pear calli were grown under continuous dark conditions at 25°C for 20 days. After 20 days of incubation, the calli were stained with phloroglucinol–HCl (Wiesner reagent) to indicate the lignin contents by a red–purple color [46]. Compared with control pear calli with red–purple color, no red–purple color of lignin staining was observed in CRISPR/Cas9-mediated *PyMYB169* or *PyNSC* knockout calli (Fig. 4G and H). As expected, the expression levels of the major lignin pathway genes, including *Py4CL1*, *PyC3H1*, *PyCSE*, *PyHCT2*, *PyCCOMT2*, *PyCAD*, *PyF5H*, *PyPOD2*, and *PyLAC1*, were reduced in *PyMYB169* or *PyNSC* knockout calli (Fig. 4I). Taking these results together, by engineering the anthocyanin and lignin biosynthesis, we demonstrate that our CRISPR/Cas9 system represents an efficient tool for loss-of-function studies, contributing to bridging the phenotype–genotype gap in pear.

### Efficient singular and multiplexed gene activation by CRISPR-Act3.0 for gain-of-function studies in pear calli

**Figure 5:**
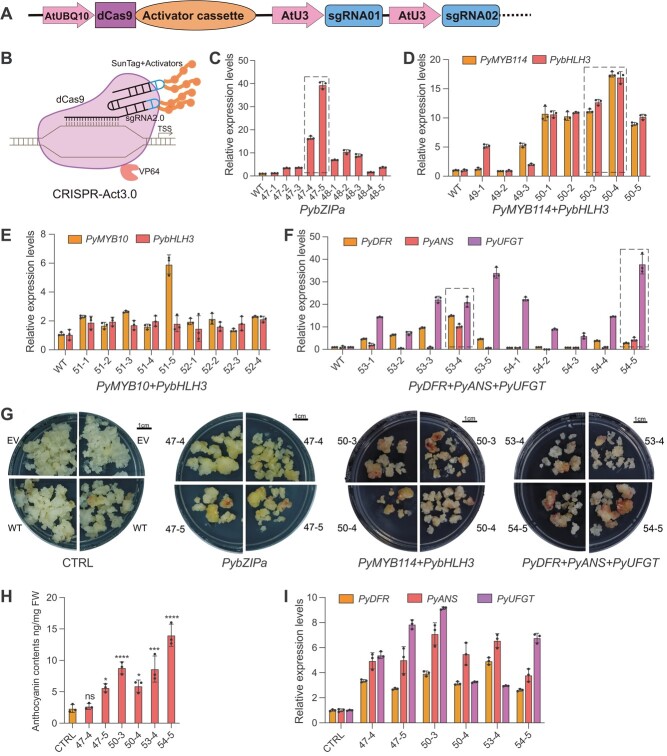
Singular and multiplexed gene activation by CRISPRa system in pear calli. (A) Schematic illustration of the CRISPRa system used in pear calli for singular and multiplexed gene activation. AtUBQ10, *Arabidopsis* ubiquitin promoter; AtU3, *Arabidopsis* U3 promoter; dCas9, deactivated zCas9. (B) Schematic diagram of the activator cassette in CRISPR-Act3.0. The dCas9 is fused with a VP64, the sgRNA2.0 scaffold contains two MS2 RNA aptamers (in blue), and SunTag+Activators contain 10 × GCN4 SunTag, a scFV, a sfGFP, and 2 × TAD activator. (C–F) CRISPRa-mediated activation of one (C), two (D, E), and three (F) anthocyanin biosynthetic genes in calli. Three to five independent transgenic lines were used in each experiment. The stable lines highlighted in the black dotted box were chosen for phenotype analysis. (G) Phenotypes of CRISPRa-mediated and control (CTRL) pear calli with continuous light treatment for 12 days. Treatment medium was MS solid medium containing 50 μM/L MeJA. (H) Anthocyanin contents of CRISPRa-mediated and control pear calli. (I) Relative transcript levels of anthocyanin biosynthetic genes in CRISPRa-mediated and control calli. WT calli and calli harboring T-DNA vector without sgRNA (EV) were used as control. *PyGAPDH* was used as the endogenous control gene. All data are presented as mean ± standard deviation (*n* = 3 independent experiments). ^ns^*P* > .05, ^*^P < .05, ^**^P < .01, ^***^P < .001, ^****^P < .0001; two-tailed Student’s *t*-test.

A third-generation CRISPRa system, CRISPR-Act3.0 [35] was adapted in this study for gene activation. Based on our findings in CRISPR/Cas9 assays, the Pol II promoter AtUBQ10 and Pol III promoter AtU3 were employed to express dCas9 and sgRNAs, respectively, for the CRISPRa system in pear (Fig. 5A). For the structure of CRISPR-Act3.0, the dCas9 was fused with an activation domain VP64, and the coupled sgRNA2.0 scaffold contained two MS2 aptamers for recruiting the MS2 bacteriophage coat protein (MCP), which was fused to the 10xGCN4 SunTag [47]. As the GCN4’s antibody, the single-chain variable fragment (scFv) fused with a super-folder green fluorescent protein (sfGFP) and 2xTAD activator can be recruited to the SunTag (Fig. 5B). Two or four different sgRNAs were designed to target the promoters of *PybZIPa*, *PyMYB10*, *PyMYB114*, *PybHLH3*, *PyDFR*, *PyANS*, or *PyUFGT*; all these genes were identified as related to anthocyanin biosynthesis. *PybZIPa*, *PyMYB10*, *PyMYB114*, and *PybHLH3* are important transcription factors involved in regulating anthocyanin biosynthesis, while *PyDFR*, *PyANS*, and *PyUFGT* are enzyme-encoding genes of the anthocyanin pathway [39, 48] (Supplementary Data Fig. S17A). Two to six sgRNAs were simultaneously expressed in each T-DNA vector for single or multiplexed gene activation (Supplementary Data Fig. S17B). The CRISPR/dCas9 components in the stable transgenic calli were detected using PCR with vector-specific primers (Supplementary Data Fig. S18). Quantitative RT–PCR analysis showed that four out of seven genes were activated at least 10-fold in some lines with specific sgRNAs (Fig. 5C–F). *PybZIPa* was activated up to 40-fold in some lines (Fig. 5C). *PyMYB114* and *PybHLH3* were simultaneously activated 10- to 20-fold in calli with the T-DNA vector pLR50 (Fig. 5D, Supplementary Data Fig. S17B), while *PyMYB10* and *PybHLH3* were only slightly activated in calli (around 2- to 6-fold) with both the T-DNA vector pLR51 and pLR52 (Fig. 5E, Supplementary Data Fig. S17B). For simultaneous *PyDFR*, *PyANS*, and *PyUFGT* activation*, PyUFGT* was activated 10- to 40-fold in most lines, whereas *PyDFR* and *PyANS* were only activated 2- to 10-fold in the same lines (Fig. 5F). These results demonstrate that CRISPR-Act3.0-mediated activation is sgRNA- or gene-specific in pear.

The stable callus lines with high levels of gene activation (highlighted by a black dotted box in Fig. 5C, D, and F) were cultured on treatment medium under continuous light conditions at 17°C for phenotype identification. The treatment medium was MS solid medium containing 50 μM/L MeJA. WT calli and calli harboring T-DNA vectors without sgRNA (EV) were used as control. After 12 days of incubation, control calli showed no or very weak red color and a low anthocyanin content (Fig. 5G and H). However, most calli with CRISPR-Act3.0-mediated *PybZIPa*, *PyMYB114*, and *PybHLH3* or *PyDFR*, *PyANS*, and *PyUFGT* activation appeared red and had a strong or moderate accumulation of anthocyanin (Fig. 5G and H), which are anticipated phenotypes for gain of function of these genes. Moreover, the expression levels of the anthocyanin pathway enzyme-encoding genes *PyDFR*, *PyANS*, and *PyUFGT* were upregulated in CRISPR-Act3.0-mediated calli compared with the control (Fig. 5I).

### Targeting specificity of CRISPR/Cas9 systems in pear calli

To assess the specificity of our CRISPR/Cas9 systems in pear, we selected five target sgRNAs for off-target analysis in stable calli (Table 1). Ten top potential off-target sites were identified by Cas-OFFinder [49]. Each potential off-target site contained two or three mismatches and was genotyped in one transgenic callus line by Sanger sequencing. Off-target mutations were detected at 2 out of 10 potential off-target sites. Both off-target sequences contained fewer than two mismatches at the PAM proximal 12-nt ‘seed’ sequence, showing 40 and 60% off-target mutation frequencies, respectively (Table 1, Supplementary Data Fig. S19). All other potential off-target sequences with two or three mismatches within the seed sequence of the protospacer had no mutations in pear calli (Table 1, Supplementary Data Fig. S19). Therefore, to ensure high targeting specificity, it is preferable to design sgRNAs whose closest off-target sites contain >2-bp mismatch nucleotides within the protospacer seed sequence.

**Table 1:** Off-target analysis of CRISPR/Cas9 system in pear calli. A total of 10 potential off-target sites were analyzed. The PAM sequence is highlighted in red and the mismatch base is highlighted in green lowercase. The off-target ratios were generated from Sanger sequencing results. The number represents mutated clones/sequenced clones

$\includegraphics{\bwartpath uhac148t1}$

## Discussion

### Highly efficient genome editing can be obtained in stable pear calli by CRISPR/Cas9 systems with the AtU3 promoter but not the AtU6 promoter

In the past decade, many efforts have been made to improve CRISPR/Cas9 editing efficiency in plants, particularly those that have a complex genome and lack an efficient stable transformation system. The editing efficiency of CRISPR/Cas9 systems could be improved by enhancing the expression level of Cas9 and sgRNAs [21]. The application of improved Pol II promoters (35S, ubiquitin, *YAO*, or the egg-cell specific promoter) and Pol III promoters (U3 or U6) is an effective strategy for enhancing the expression of Cas9 and sgRNAs in plants [18, 19, 21]. For example, the CRISPR/Cas9 system with PcUbi4-2 expressing Cas9 and MdU3/U6 expressing sgRNAs led to higher editing efficiency (84–93%) in apple [37]. The CRISPR/Cas9 system with the 35S promoter expressing Cas9 and AtU6-1 expressing sgRNAs only generated moderate editing efficiency (31.8%) in apple [50]. For sgRNA expression, both the AtU6-26 promoter and the FveU6-2 promoter showed high-efficiency genome editing in strawberry [51]. The sgRNAs driven by VvU3/U6 promoters resulted in higher editing efficiency in grape cells (14.65–22.10%) than sgRNAs driven by the AtU6 promoter (13.67%) [21].

Application of CRISPR/Cas systems in pear was challenging because of the highly heterozygous genome and inefficient stable transformation method [36, 37]. In this study, we used the maize codon-optimized Cas9, which is highly efficient for genome editing in *Arabidopsis* [52], rice [53], maize [54], wheat [55], tomato [56], and poplar [20]. We compared four different CRISPR/Cas9 systems containing two Pol II promoters (35S and AtUBQ10) for Cas9 expression and two Pol III promoters (AtU3 and AtU6) for sgRNA expression in stable pear calli (Fig. 1A). We found that both the 35S and the AtUBQ10 promoter induced extremely high editing efficiencies (nearly 100%) when sgRNAs were driven by the AtU3 promoter (Fig. 1F and G). By contrast, poor editing efficiencies were observed with the AtU6 promoter expressing sgRNAs, regardless the promoters for Cas9 expression (Fig. 1C–E). These results demonstrated that the AtU3 promoter is more efficient than the AtU6 promoter for CRISPR/Cas9 systems in pear. This is consistent with the recent observations that AtU3 outperforms AtU6 for Cas9-mediated genome editing in tomato [56] and poplar [20]. Our established CRISPR/Cas9 system resulted in high editing efficiencies (nearly 100%) in pear calli.

Because of the bottlenecks in tissue culture and stable transformation, only proof of concept for CRISPR/Cas9 gene editing had been conducted in fruit crops, mainly based on the gene *PDS* rather than trait-related genes, such as in citrus [57], apple [50], grape [58], kiwifruit [59], banana [60, 61], strawberry [62, 63], and pear [37]. Recently, increasing numbers of CRISPR studies have focused on improving editing efficiency, enhancing resistance to biotic and abiotic stresses, and promoting early flowering and dwarfing, and gene functional studies have been reported in fruit crops [9, 21, 64, 65]. However, to our knowledge, our CRISPR/Cas9 system developed in pear represents the most efficient CRISPR system in fruit tree crops: 93% in apple [37], 43.24% in grape [21], 64.7% in citrus [66], and 75% in kiwifruit [9]. Hence, we have a highly efficient CRISPR/Cas9 system that may be applicable to other fruit tree crops.

### CRISPR/Cas12a and Cas12b have low efficiency for genome editing in pear

The canonical CRISPR/Cas9 recognizes an NGG PAM [22], while CRISPR/Cas12a and Cas12b prefer T-rich PAMs [23, 24], contributing to an expanded targeting scope. Importantly, the CRISPR/Cas12a and Cas12b systems have shown comparable activity and higher targeting specificity compared with CRISPR/Cas9 in many plant species, such as rice [24, 25, 28, 67], maize [54], cotton [68, 69], and citrus [70]. It is worth noting that the aforementioned species are general thermophilic crops, which need relatively high temperatures (>30°C) for tissue culture and growth. Previous studies demonstrated that both Cas12a and Cas12b nucleases are temperature-sensitive and require a temperature of >28°C for high activity [71, 72]. Therefore, the CRISPR/Cas12a and Cas12b systems induced relatively low editing efficiency in dicotyledons that typically grow at temperatures of 25°C or lower. For example, CRISPR/LbCas12a had no editing activity at 22°C in *Arabidopsis*, but the editing efficiency increased to 35% under 29°C treatment for 1 month [71]. In this study, we found that both CRISPR/Cas12a and Cas12b systems, including LbCas12a, Mb2Cas12a, and AaCas12b, had low efficiency for editing in pear calli cultured at 25°C (Fig. 3B–E, G, and H). Their editing efficiencies were not induced or only slightly enhanced after stable pear calli were incubated at 30°C for 7 days (Fig. 3B–E, G, and H). These results indicate that a long-term high-temperature treatment might be critical for improving the editing efficiencies of CRISPR/Cas12a and Cas12b systems in pear. However, the pear calli could not survive under long-period high-temperature conditions, preventing us from further optimizing CRISPR/Cas12a and Cas12b systems in pear. In the future, temperature-insensitive Cas12a and Cas12b variants need to be developed for genome editing in pear.

### The potent CRISPR/Cas9 system is a powerful tool for gene loss-of-function studies in pear

Targeted mutagenesis by genome editing tools such as CRISPR/Cas9 is an efficient approach to probing the causal relationships between genotype and phenotype in plants. However, the application of CRISPR/Cas systems in fruit crops is still in its infancy due to the high levels of genomic heterozygosity and challenges in tissue culture and stable transformation [7, 73]. Callus is a good system for such reverse genetics analysis in fruit trees as it bypasses the lengthy plant regeneration and juvenile stage. In addition, the efficiency of stable transformation of callus is usually very high, and transgenic calli can be obtained with a short screening time, which could accelerate gene function studies in fruit crops. In this study we showed highly efficient multiplexed editing (nearly 100%) of anthocyanin and lignin biosynthetic genes by assembling multiple sgRNA expression cassettes into single T-DNA vectors (Fig. 4B). The phenotypes of pear calli were consistent with genotyping results (Fig. 4D and G), suggesting that the established CRISPR/Cas9 system allows rapid loss-of-function analysis in pear. Recently, CRISPR/Cas9 systems-mediated high-throughput functional genomics screening has been implemented in several crops, including rice [74, 75], tomato [76], maize [77], and soybean [78]. The high editing efficiency of our CRISPR/Cas9 system would enable us to perform high-throughput functional genomics studies in pear. Undoubtedly, our CRISPR/Cas9 system will advance the understanding of various developmental processes, such as anthocyanin, lignin, sugar, acid, and aroma accumulation, as well as biotic and abiotic stress responses, which could be fully studied using pear calli. This study also provides a valuable model for other species without a stable plant transformation system. At the same time, genome editing using a callus system has its own limitations. For example, some specific types of phenotype research, such as dwarfing and flowering, are unachievable using a callus system. But it is conceivable that an embryogenic callus may be used if regeneration of the whole plant is needed for phenotypic analysis.

### The CRISPRa system is a powerful tool for gain-of-function studies in pear

Calli have been widely used in gene functional studies in fruit crops mainly via the conventional gene overexpression approach [41–43, 79, 80], due to its high efficiency and fast detection in transgenic lines. CRISPRa systems outperformed the conventional gene overexpression approach, due to the convenience of expressing multiple sgRNAs [32, 35]. In this study, we achieved efficient singular and multiplexed gene activation with CRISPR-Act3.0 using the AtUBQ10 and AtU3 promoters. Consistent with the previous observation in rice [35], different sgRNAs targeting the same gene could result in large variation of activation efficiency, suggesting the significance of sgRNA design (Fig. 5C–F, Supplementary Data Fig. S17). Furthermore, CRISPRa-induced transcriptional activation is easier for some genes than for others, which might result from the discrepant transcriptional control mechanisms imposed on different genes. Nevertheless, our results suggest the CRISPRa system is a powerful tool for gene activation that can render phenotypic changes as gain of function in pear (Fig. 5G). However, it is recommended that multiple sgRNAs are assessed for each target gene to identify the most potent sgRNAs. With our stable callus system, it is possible to fast-screen sgRNA activity for activation, which will ultimately help achieve high-level gene activation in pear.

In conclusion, we have established a highly efficient CRISPR/Cas9 system for genome editing in pear, which is preferred over CRISPR/Cas12a and Cas12b systems. We successfully applied it to gene loss-of-function studies using a pear callus system. Furthermore, we demonstrated efficient singular and multiplexed gene activation by CRISPR-Act3.0. Overall, this study provided a CRISPR toolbox that will aid loss-of-function and gain-of-function research in pear and potentially other fruit crops.

## Materials and methods

### Plant materials and growth conditions

Dedifferentiated pear calli were induced from *Pyrus communis* based on a previous report [42]. The calli were cultured on MS solid medium containing 1.0 mg/L 2,4-dichlorophenoxyacetic acid (2,4-D) and 0.5 mg/L N6-benzyladenine (6-BA) under continuous dark conditions at 25°C and subcultured every 2 weeks. Pear plants were grown on MS solid medium containing 1.0 mg/L 6-BA and 0.2 mg/L indole-3-butytric acid (IBA) at 25°C with a 16-hour light/8-hour dark photoperiod and subcultured every month.

### Vector construction

All T-DNA expression vectors were constructed based on a Golden Gate cloning and three-way Gateway cloning system as previously described [24, 28, 35]. Briefly, each sgRNA of the target gene was first cloned into sgRNA expression cassette pYPQ131, pYPQ132, pYPQ133, or pYPQ134 by T4 DNA ligase. Then, multiple sgRNA cassettes were assembled into sgRNA expression vector pYPQ142 or pYPQ143 or pYPQ144 by Golden Gate reactions to simultaneously express multiple sgRNAs in one T-DNA vector. Finally, the sgRNA expression vector containing multiple sgRNAs and CRISPR-Cas9/dCas9/Cas12a/Cas12b expression cassette were cloned into the destination backbone vector pYPQ202 (hygromycin resistance) or pCGS710 (kanamycin resistance) to generate the final T-DNA expression vectors (such as pLR01 and pLR02) by three-way LR reaction. All backbone vectors used in this study are available from Addgene: pYPQ131A (no. 69273), pYPQ132A (no. 69274), pYPQ131B (no. 69281), pYPQ132B (no. 69282), pYPQ131-STU-Fn (no. 138095), pYPQ132-STU-Fn (no. 138098), pYPQ133-STU-Fn (no. 138101), pYPQ134-STU-Fn (no. 138104), pYPQ131-STU-Lb (no. 138096), pYPQ132-STU-Lb (no. 138099), pYPQ133-STU-Lb (no. 138102), pYPQ134-STU-Lb (no. 138105), pYPQ131B2.0 (no. 99885), pYPQ132B2.0 (no. 99888), pYPQ133B2.0 (no. 99892), pYPQ142 (no. 69294), pYPQ143 (no. 69295), pYPQ144 (no. 69296), pYPQ141-ZmUbi-RZ-Aac (no. 129685), pYPQ144-ZmUbi-pT (no. 138108), pYPQ166 (no. 109328), pYPQ230 (no. 86210), pYPQ284 (no. 138116), pYPQ292 (no. 129672), pYPQ-dzCas9-Act3.0 (no. 158414), and pYPQ202 (no. 86198).

### Stable transformation of pear calli and pear plants

For the stable transformation assays, the final T-DNA expression vectors (such as pLR01 and pLR02) were transformed into *Agrobacterium tumefaciens* strain GV3101 using the freeze–thaw method, according to the manufacturer’s instructions (Weidi, http://www.weidibio.com). For stable transformation of pear calli, *Agrobacterium* cells were resuspended in MS liquid medium with 100 mM acetosyringone AS to an OD_600_ of 0.6–0.8, and then incubated at room temperature with slow shaking for 1 hour. Dedifferentiated pear calli were then incubated in the *Agrobacterium* suspension for 15 minutes. The T-DNA vector without sgRNA (EV) was transformed as negative control. After infiltration, calli were incubated on MS solid medium with 100 mM AS under dark conditions at 25°C for 2 days. Then calli were transferred to MS solid medium with 15 mg/L hygromycin B and 150 mg/L cefotaxime for transgenic callus selection. After a month, the regenerated calli were collected and subcultured every 15 days for genotyping and phenotyping. During callus regeneration, a mass of regenerated calli (highlighted by an orange dotted circle in Fig. 1B), is considered to be an independent stable line (an independent stable line always contains more than one single cell).

For stable transformation of pear plants, leaves were wounded with a scalpel and preincubated on NN69 (Nitsch and Nitsch, 1969) solid medium containing 3.0 mg/L thidiazuron (TDZ) and 0.3 mg/L IBA under dark conditions at 25°C for 5 days. *Agrobacterium* cells were resuspended in NN69 liquid medium with 100 mM AS to an OD_600_ of 0.6. The pre-wounded leaves were dipped into the inoculum with slow shaking for 20 minutes, followed by co-culturing on NN69 solid medium containing 3.0 mg/L TDZ, 0.3 mg/L IBA, and 100 mM AS under dark conditions at 25°C for 2 days. At the end of co-culture, leaves were plated on NN69 solid medium containing 3.0 mg/L TDZ, 0.3 mg/L IBA, and 150 mg/L cefotaxime at 25°C under continuous dark conditions. Note that the leaves were always plated abaxial side down on solid medium. One month later, the regenerated plants were transferred to growth medium containing 150 mg/L cefotaxime and 3 mg/L hygromycin B and subcultured every month for genotyping and phenotyping.

### Mutation analysis by PCR–RFLP, Sanger sequencing, and high-throughput sequencing

Pear calli were collected for genomic DNA extraction using the CTAB (cetyl trimethylammonium bromide) method. The genomic regions flanking the target sites were PCR-amplified for PCR–RFLP analysis, Sanger sequencing, and high-throughput sequencing. First, the CRISPR/Cas components in the stable transgenic calli were detected using PCR with vector-specific primers. Then, Cas nuclease-induced mutations were detected by PCR–RFLP followed by Sanger sequencing or high-throughput sequencing. For PCR–RFLP analysis, PCR amplicons were digested with corresponding restriction enzymes and visualized on 1.5% TAE (Tris, acetic acid, EDTA) agarose gels. ImageJ (https://imagej.nih.gov/ij/) was used to quantify the mutation frequencies. PCR amplicons were cloned into the pMD19-T vector for Sanger sequencing. Editing frequencies were calculated as the number of mutated clones divide by the total number of sequenced clones. For high-throughput amplicon deep sequencing, PCR amplicons with sequencing barcodes were sent to Novogene for quality check and followed by high-throughput sequencing using an Illumina HiseqX-PE150 platform. Clean sequencing data were analyzed by CRISPRMatch [81] for mutation frequencies and profiles. The total mutation ratio at each target site was calculated by dividing the number of mutant reads (include deletion and insertion) by the total number of reads. For off-target analysis, Cas-OFFinder [49] was used to identify potential off-target sites. To detect possible mutations, PCR amplicons were also cloned into the pMD19-T vector for Sanger sequencing.

### RNA extraction and qRT–PCR analysis

Total RNA was extracted from transgenic calli with the Plant Total RNA Isolation Plus Kit according to the manufacturer’s instructions (Foregene, http://www.foregene.com). First-strand cDNA was synthesized with the EasyScript^®^ First-Strand cDNA Synthesis SuperMix Kit and the qRT–PCR assay was conducted with TransStart^®^ Green qPCR SuperMix (Transgene, https://www.transgen.com.cn/rt_pcr.html) using a real-time LightCycler 480 II^®^ PCR detection system (Roche). The transcript expression levels were determined by the 2^-ΔΔCt^ method. *PyGAPDH* was used as the endogenous control gene. All primers used in this study are listed in Supplementary Data Table S1.

### Phenotyping, extraction, and measurement of anthocyanin and lignin

Based on the different purposes, different culture conditions were employed in phenotyping analysis. For CRISPR/Cas9-mediated *PyMYB10* and *PyMYB114* knockout calli, the stable pear calli were cultured on MS solid medium without nitrogen but containing 200 μM/L MeJA under continuous light conditions at 17°C for 7 days before anthocyanin analysis. For CRISPRa-mediated activation of anthocyanin biosynthetic genes in calli, stable pear calli were cultured on MS solid medium containing 50 μM/L of MeJA under continuous light conditions at 17°C for 12 days before anthocyanin analysis. Total anthocyanin was extracted and measured as described in our previous report [39].

For CRISPR/Cas9-mediated *PyMYB169* or *PyNSC* knockout calli, stable pear calli were cultured on MS solid medium containing 100 μM/L brassinolide but no 2,4-D and 6-BA under continuous dark conditions at 25°C for 20 days before lignin analysis [82]. Total lignin was extracted and measured as described in our previous report [44]. Note that there is no lignin biosynthesis in pear calli if 2,4-D and 6-BA are added to the MS solid medium.

## Acknowledgements

This work was supported by grants from the National Science Foundation of China (31820103012, 31725024), the National Key Research and Development Program (2018YFD1000200), the Earmarked Fund for China Agriculture Research System (CARS-28), and the Earmarked Fund for Jiangsu Agricultural Industry Technology System JATS [2021]453. This work was also supported by the National Science Foundation Plant Genome Research Program grant (IOS-1758745) to Y.Q. H.L, J.C., and R.T. were supported by a scholarship from the National Training Program of Innovation and Entrepreneurship for Undergraduates (202110307017). We thank the Centre of Pear Engineering Technology Research, State Key Laboratory of Crop Genetics and Germplasm Enhancement, Nanjing Agricultural University for supporting this project.

## Author contributions

J.W. and M.M. designed the experiments. J.W. and Y.Q. supervised the research. M.M, C.P., and Y.Z. generated all the vectors. M.M., H.L., Z.Y., J.C., R.T., and J.L. performed the stable transformation of pear calli and plants. M.M. did all genotyping and phenotyping analysis with the help of C.S. and Y.X. J.W., Y.Q., and M.M. wrote the paper with input from other authors. All authors read and approved the final manuscript.

## Data availability

The high-throughput sequencing data sets have been submitted to the National Center for Biotechnology information (NCBI) database under Sequence Read Archive (SRA) BioProject ID PRJNA787753.

## Conflict of interest

The authors declare that they have no conflicts of interest.

## Supplementary data

Supplementary data is available at *Horticulture Research* online.

## Supplementary Material

Web_Material_uhac148
