# Supplementary material for: Highly efficient CRISPR systems for loss-of-function and gain-of-function research in pear calli
Source: Hortic Res. 2022 Jun 30;9:uhac148. doi: 10.1093/hr/uhac148 (PMC9437716; doi:10.1093/hr/uhac148)
Supplement: Web_Material_uhac148 [file web_material_uhac148.zip › Supplemental Figures-2022.6.21.pdf]

Figure S1. DNA sequence alignment of *PyPDS*. Ref, reference. DS, ‘Dangshansuli’ pear fruits. Callus, dedifferentiated ‘Clapp’s Favorite’ pear calli. Qiuzili, pear plants for tissue culture.

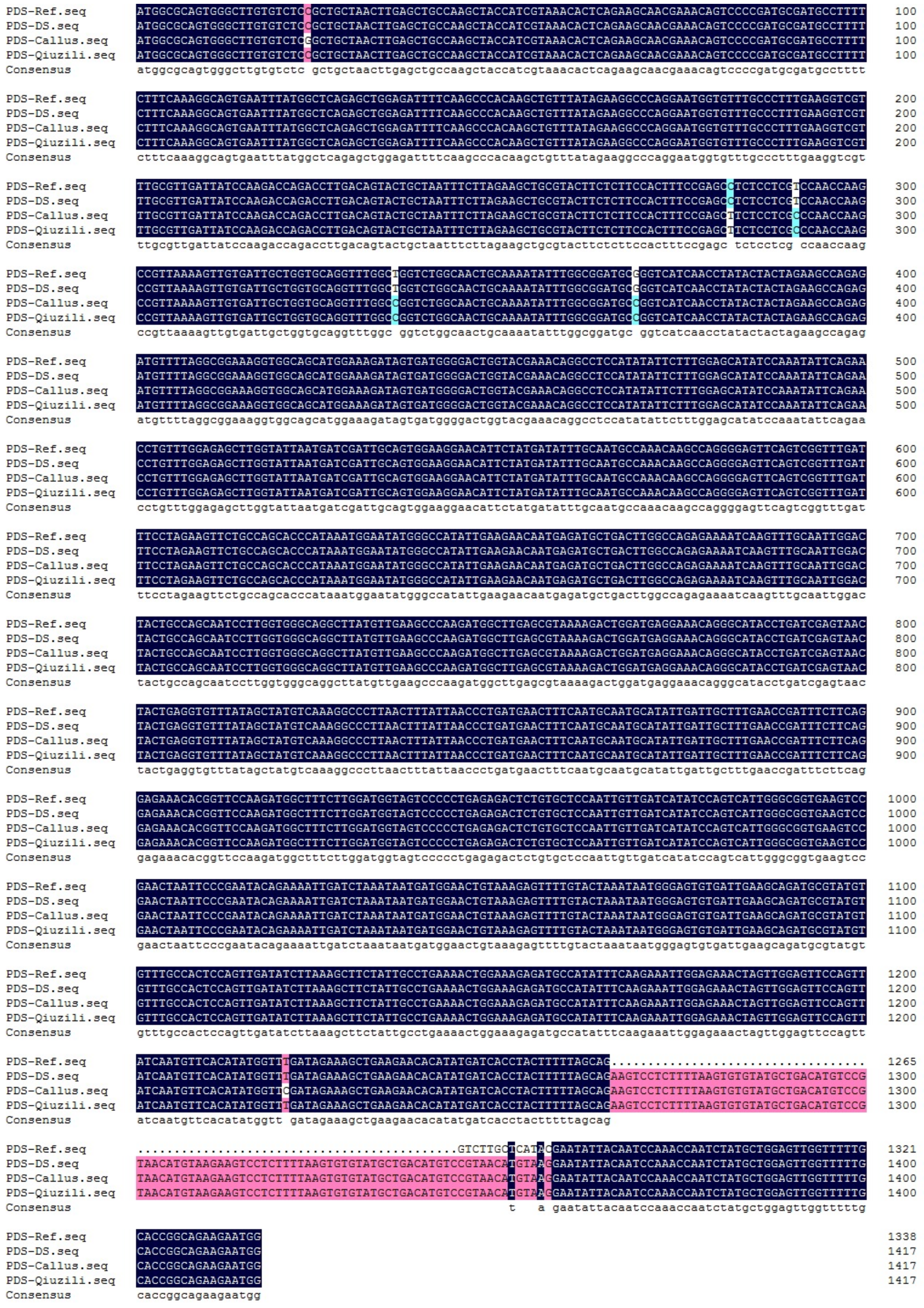

Figure S2. DNA sequence alignment of *PyGID1.* Ref, reference. DS, ‘Dangshansuli’ pear fruits. Callus, dedifferentiated ‘Clapp’s Favorite’ pear calli. Qiuzili, pear plants for tissue culture.

```
GID1-Ref.seq       ATGTGTTGGGTTTTCTTCAAGCTTAGATTTTGGAGTGGAAATTCTTCAAAACCGGTTTCCATGGCTGGGGGCAATGAAGTCAACGTAAATGAATCCAGGA   100
GID1-DS.seq        ATGTGTTGGGTTTTCTTCAAGCTTAGATTTTGGAGTGGAAATTCTTCAAAACCGGTTTCCATGGCTGGGGGCAATGAAGTCAACGTAAATGAATCCAGGA   100
GID1-Callus.seq    ATGTGTTGGGTTTTCTTCAAGCTTAGATTTTGGAGTGGAAATTCTTCAAAACCGGTTTCCATGGCTGGGGGCAATGAAGTCAACGTAAATGAATCCAGGA   100
GID1-Qiuzili.seq   ATGTGTTGGGTTTTCTTCAAGCTTAGATTTTGGAGTGGAAATTCTTCAAAACCGGTTTCCATGGCTGGGGGCAATGAAGTCAACGTAAATGAATCCAGGA   100
Consensus          atgtgttgggttttcttcaagcttagattttggagtggaaattcttcaaaaccggtttccatggctgggggcaatgaagtcaacgtaaatgaatccagga

GID1-Ref.seq       CAGTGGTTCCACTGAATACATGGGTCCTCATCTCAAATTTCAAGCTGTCATACAATCTTCTGCGCCGCCCTGACGGGACTTTCAACCGGCACTTGGCGGA   200
GID1-DS.seq        CAGTGGTTCCACTGAATACATGGGTCCTCATCTCAAATTTCAAGCTGTCATACAATCTTCTGCGCCGCCCTGACGGGACTTTCAACCGGCACTTGGCGGA   200
GID1-Callus.seq    CAGTGGTTCCACTGAATACATGGGTCCTCATCTCGAATTTCAAGCTGTCATACAATCTTCTACGCCGCCCTGACGGGACTTTCAACCGCCACTTGGCGGA   200
GID1-Qiuzili.seq   CAGTGGTTCCACTGAATACATGGGTCCTCATCTCGAATTTCAAGCTGTCATACAATCTTCTGCGCCGCCCTGACGGGACTTTCAACCGGCACTTGGCGGA   200
Consensus          cagtggttccactgaatacatgggtcctcatctc aatttcaagctgtcatacaatcttct cgccgccctgacgggactttcaaccg cacttggcgga

GID1-Ref.seq       GTTCCTTGATCGGAAAGTGCCAGCCAATGCTAACCCCGTTGATGGGGTTGTCTCGTTTGATGTCATCATCGATCGCGAAACTAGCCTGCTAAGTCGAATC   300
GID1-DS.seq        GTTCCTTGATCGGAAAGTGCCAGCCAATGCTAACCCCGTTGATGGGGTTGTCTCGTTTGATGTCATCATCGATCGCGAAACTAGCCTGCTAAGTCGAATC   300
GID1-Callus.seq    GTTCCTTGATCGGAAAGTGCCAGCCAATGCTAACCCCGTTGATGGGGTTGTCTCGTTTGATGTCATCATCGACCGCGAAACTAGCCTGCTAAGTCGAATC   300
GID1-Qiuzili.seq   GTTCCTTGATCGGAAAGTGCCAGCCAATGCTAACCCCGTTGATGGGGTTGTCTCGTTTGATGTCATCATCGATCGCGAAACTAGCCTGCTAAGTCGAATC   300
Consensus          gttccttgatcggaaagtgccagccaatgctaaccccgttgatggggttgtctcgtttgatgtcatcatcga cgcgaaactagcctgctaagtcgaatc

GID1-Ref.seq       TATCATCCAGACAATGCTGATCTATCCCCGCTGAACATTGTTGATCTTAAGAGACCTGTGAACAAGGAGGTTCTTCCTGTCATAGTTTTCTTCCATGGTG   400
GID1-DS.seq        TATCATCCAGACAATGCTGATCTATCCCCGCTGAACATTGTTGATCTTAAGAGACCTGTGAACAAGGAGGTTCTTCCTGTCATAGTTTTCTTCCATGGTG   400
GID1-Callus.seq    TATCATCCAGACAATGCTGATCTATCCCCGCTGAACATTGTTGATCTTAAGAGACCTGTGAACAAGGAAGTTCTTCCTGTCATAGTTTTCTTCCATGGTG   400
GID1-Qiuzili.seq   TATCATCCAGACAATGCTGATCTATCCCCGCTGAACATTGTTGATCTTAAGAGACCTGTGAACAAGGAGGTTCTTCCTGTCATAGTTTTCTTCCATGGTG   400
Consensus          tatcatccagacaatgctgatctatccccgctgaacattgttgatcttaagagacctgtgaacaagga gttcttcctgtcatagttttcttccatggtg

GID1-Ref.seq       GGAGCTTTGTACACTCCTCTTCAAACAGCGGTATTTATGATATTCTGTGCCGCCGACTAGTTGGTATCTGCAAGGCTGTAGTGGTCTCTGTGAACTATCG   500
GID1-DS.seq        GGAGCTTTGTACACTCCTCTTCAAACAGCGGTATTTATGATATTCTGTGCCGCCGACTAGTTGGTATCTGCAAGGCTGTAGTGGTCTCTGTGAACTATCG   500
GID1-Callus.seq    GGAGCTTTGTACACTCCTCTTCAAACAGCGGTATTTATGATATTCTGTGCCGCCGACTAGTTGGTATCTGCAAAGCTGTAGTGGTCTCTGTGAACTATCG   500
GID1-Qiuzili.seq   GGAGCTTTGTACACTCCTCTTCAAACAGCGGTATTTATGATATTCTGTGCCGCCGACTAGTTGGTATCTGCAAGGCTGTAGTGGTCTCTGTGAACTATCG   500
Consensus          ggagctttgtacactcctcttcaaacagcggtatttatgatattctgtgccgccgactagttggtatctgcaa gctgtagtggtctctgtgaactatcg

GID1-Ref.seq       CCGGGCACCTGAAAACCGCTATCCATGTGCCTATGATGATGGGTGGACAGCCCTGAAGTGGGTGAAGTCTAGACCATGGCTTAAAAGTACGAAGGACTCA   600
GID1-DS.seq        CCGGGCACCTGAAAACCGCTATCCATGTGCCTATGATGATGGGTGGACAGCCCTGAAGTGGGTGAAGTCTAGACCATGGCTTAAAAGTACGAAGGACTCA   600
GID1-Callus.seq    CCGGGCACCTGAAAACCGCTATCCATGTGCCTATGATGATGGTTGGACAGCCCTGAAGTGGGTGAAGTCTAGACCATGGCTTAAAAGTACGAAGGACTCA   600
GID1-Qiuzili.seq   CCGGGCACCTGAAAACCGCTATCCATGTGCCTATGATGATGGGTGGACAGCCCTGAAGTGGGTGAAGTCTAGACCATGGCTTAAAAGTACGAAGGACTCA   600
Consensus          ccgggcacctgaaaaccgctatccatgtgcctatgatgatgg tggacagccctgaagtgggtgaagtctagaccatggcttaaaagtacgaaggactca

GID1-Ref.seq       AAAGTTCATATCTATCTCGCTGGCGATAGCTCGGGTGGGAACATTGTACACAATGTTGCTTTAAGAGCTGCAGAATCTGGAATCAATGTATTGGGAAATA   700
GID1-DS.seq        AAAGTTCATATCTATCTCGCTGGCGATAGCTCGGGTGGGAACATTGTACACAATGTTGCTTTAAGAGCTGCAGAATCTGGAATCAATGTATTGGGAAATA   700
GID1-Callus.seq    AAAGTTCATATCTATCTCGCTGGCGATAGCTCGGGTGGGAACATTGTACACAATGTTGCTTTAAGAGCTGCAGAATCTGGAATCAATGTATTGGGAAATA   700
GID1-Qiuzili.seq   AAAGTTCATATCTATCTCGCTGGCGATAGCTCGGGTGGGAACATTGTACACAATGTTGCTTTAAGAGCTGCAGAATCTGGAATCAATGTACTGGGAAATA   700
Consensus          aaagttcatatctatctcgctggcgatagctcgggtgggaacattgtacacaatgttgctttaagagctgcagaatctggaatcaatgta tgggaaata

GID1-Ref.seq       TACTGCTCAACCCTATGTTTGGTGGGCAGGAGCGGACTGAATCCGAGATGCGATTGGATGGGAAATACTTTGTCACCATCCAAGACCGGGACTGGTACTG   800
GID1-DS.seq        TACTGCTCAACCCTATGTTTGGTGGGCAGGAGCGGACTGAATCCGAGATGCGATTGGATGGGAAATACTTTGTCACCATCCAAGACCGGGACTGGTACTG   800
GID1-Callus.seq    TACTGCTCAACCCTATGTTTGGTGGGCAGGAGCGGACTGAATCCGAGATGCGATTGGATGGGAAATACTTTGTCACCATCCAAGACCGGGACTGGTACTG   800
GID1-Qiuzili.seq   TACTGCTCAACCCTATGTTTGGTGGGCAGGAGCGGACTGAATCCGAGATGCGATTGGATGGGAAATACTTTGTCACCATCCAAGACCGGGACTGGTACTG   800
Consensus          tactgctcaaccctatgtttggtgggcaggagcggactgaatccgagatgcgattggatgggaaatactttgtcaccatccaagaccgggactggtactg

GID1-Ref.seq       GAGAGCTTTACTCCCTGAGGGAGAAGATAGGGACCATCCAGCATGTAACCCATTTGGTCCCCGGGGTCAAAGCCTCGAAGCTGTCAAGTTCCCGAAGAGC   900
GID1-DS.seq        GAGAGCTTTACTCCCTGAGGGAGAAGATAGGGACCATCCAGCATGTAACCCATTTGGTCCCCGGGGTCAAAGCCTCGAAGCTGTCAAGTTCCCGAAGAGC   900
GID1-Callus.seq    GAGAGCTTTACTCCCTGAGGGAGAAGATAGGGACCATCCAGCATGTAACCCATTTGGTCCCCGGGGTCAAAGCCTCGAAGCTGTCAAGTTCCCGAAGAGC   900
GID1-Qiuzili.seq   GAGAGCTTTACTCCCTGAGGGAGAAGATAGGGACCATCCAGCATGTAACCCATTTGGTCCCCGGGGTCAAAGCCTCGAAGCTGTCAAGTTCCCGAAGAGC   900
Consensus          gagagctttactccctgagggagaagatagggaccatccagcatgtaacccatttggtccccggggtcaaagcctcgaagctgtcaagttcccgaagagc

GID1-Ref.seq       CTTGTTGTGGTGGCTGGTTTGGATCTTGTTCAGGACTGGCAATTGGCTTATGCTAGAGGGCTCGAGAGGGCTGGCATAAACATGAAACTCATGTATCTTG  1000
GID1-DS.seq        CTTGTTGTGGTGGCTGGTTTGGATCTTGTTCAGGACTGGCAATTGGCTTATGCTAGAGGGCTCGAGAGGGCTGGCATAAACATGAAACTCATGTATCTTG  1000
GID1-Callus.seq    CTTGTTGTGGTGGCTGGTTTGGATCTTGTTCAGGACTGGCAATTGGCTTATGCTAGAGGGCTCGAGAGGGCTGGCATAAACATGAAACTCATGTATCTTG  1000
GID1-Qiuzili.seq   CTTGTTGTGGTGGCTGGTTTGGATCTTGTTCAGGACTGGCAATTGGCTTATGCTAGAGGGCTCGAGAGGGCTGGCATAAACATGAAACTCATGTATCTTG  1000
Consensus          cttgttgtggtggctggtttggatcttgttcaggactggcaattggcttatgctagagggctcgagagggctggcataaacatgaaactcatgtatcttg

GID1-Ref.seq       AGCAGGCCACAATTGGTTTCTACCTGTTGCCGAATAATGAGCATTTCTACACCGTGATGGACGAGATCAGTAAATTCGTGTGCTCCAACTATTG        1094
GID1-DS.seq        AGCAGGCCACAATTGGTTTCTACCTGTTGCCGAATAATGAGCATTTCTACACCGTGATGGACGAGATCAGTAAATTCGTGTGCTCCAACTATTG        1094
GID1-Callus.seq    AGCAGGCCACAATTGGTTTCTACCTGTTGCCGAATAATGAGCATTTCTACACCGTGATGGACGAGATCAGTAAATTCGTGTGCTCCAACTATTG        1094
GID1-Qiuzili.seq   AGCAGGCCACAATTGGTTTCTACCTGTTGCCGAATAATGAGCATTTCTACACCGTGATGGACGAGATCAGTAAATTCGTGTGCTCCAACTATTG        1094
Consensus          agcaggccacaattggtttctacctgttgccgaataatgagcatttctacaccgtgatggacgagatcagtaaattcgtgtgctccaactattg
```

Figure S3. DNA sequence alignment of *PyTFL1.1/1.2.* REF, reference. DS, 'Dangshansuli' pear fruits. Callus, dedifferentiated 'Clapp's Favorite' pear calli. Qiuzili, pear plants for tissue culture.

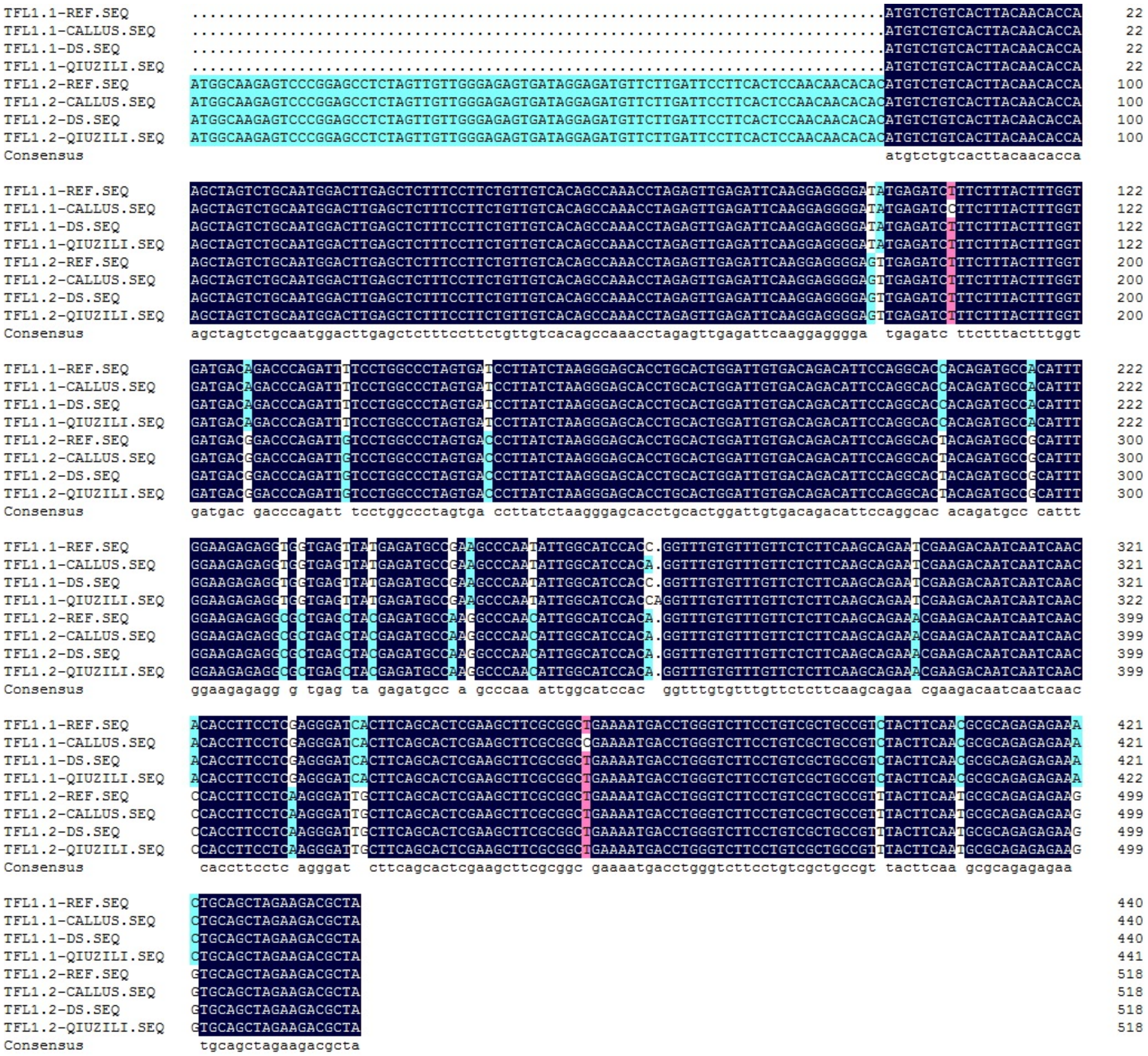

Figure S4. Guide RNA design and construction of CRISPR/Cas9 vectors for genome editing. A, Schematics of the sgRNA positions and sequences. *PyPDS, PyGID1* and *PyTFL1.1/1.2* are target genes for genome editing. The 20 nt protospacers with an NGG PAM are designed for Cas9. B, Vectors of different CRISPR/Cas9 systems for genome editing.

A

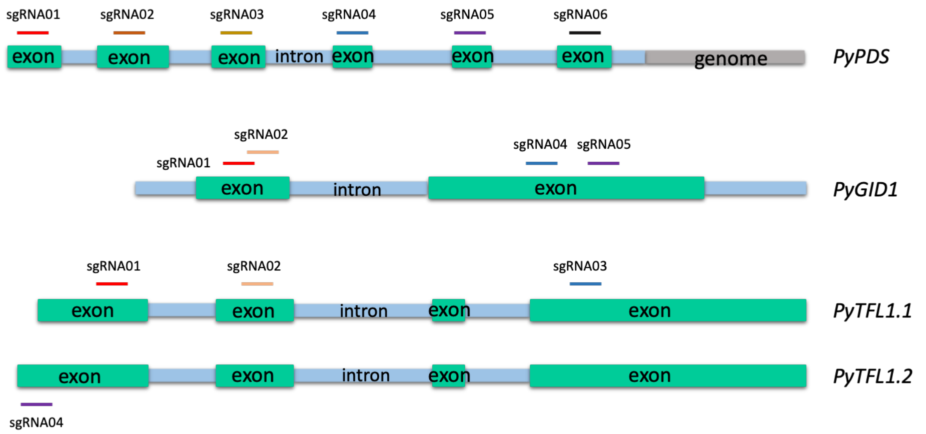

Target genes	sgRNAs	Target sequences	PAM
PyPDS	sgRNA01	AAGCTGTTTATAGAAGGCCC	AGG
	sgRNA02	AGAAATTAGCAGTACTGTCA	AGG
	sgRNA03	TGCAAAATATTTGGCGGATG	CGG
	sgRNA04	TGCAATGCCAAACAAGCCAG	GGG
	sgRNA05	GATCATATCCAGTCATTGGG	CGG
	sgRNA06	GGCAACTGCAAAATATTTGG	CGG
PyGID1	sgRNA01	TCAAAACCGGTTTCCATGGC	TGG
	sgRNA02	TTGCCCCCAGCCATGGAAAC	CGG
	sgRNA04	GATAGCGGTTTTCAGGTGCC	CGG
	sgRNA05	CTCGCTGGCGATAGCTCGGG	TGG
PyTFL1.1/1.2	sgRNA01	CTAGAGTTGAGATTCAAGGA	GGG
	sgRNA02	TGCAGGTGCTCCCTTAGATA	AGG
	sgRNA03	GAGATGCCGAAGCCCAATAT	TGG
	sgRNA04	GTGACAGACATGTGTGTTGT	TGG

zCas9 [sgRNA: 20nt + PAM: NGG]

B

Vectors	Target genes	sgRNA		Cas9	Resistance
pLR01	*PyPDS*	AtU6-gRNA01	AtU6-gRNA03	AtUBQ10-zCas9	Hygromycin
pLR02	*PyPDS*	AtU3-gRNA01	AtU3-gRNA03	AtUBQ10-zCas9	Hygromycin
pLR03	*PyPDS*	AtU6-gRNA02	AtU6-gRNA04	AtUBQ10-zCas9	Hygromycin
pLR04	*PyPDS*	AtU3-gRNA02	AtU3-gRNA04	AtUBQ10-zCas9	Hygromycin
pLR05	*PyPDS*	AtU6-gRNA05	AtU6-gRNA06	AtUBQ10-zCas9	Hygromycin
pLR06	*PyPDS*	AtU3-gRNA05	AtU3-gRNA06	AtUBQ10-zCas9	Hygromycin
pLR07	*PyGID1*	AtU6-gRNA02	AtU6-gRNA04	AtUBQ10-zCas9	Hygromycin
pLR08	*PyGID1*	AtU3-gRNA02	AtU3-gRNA04	AtUBQ10-zCas9	Hygromycin
pLR09	*PyGID1*	AtU6-gRNA01	AtU6-gRNA05	AtUBQ10-zCas9	Hygromycin
pLR10	*PyGID1*	AtU3-gRNA01	AtU3-gRNA05	AtUBQ10-zCas9	Hygromycin
pLR11	*PyTFL1.1/1.2*	AtU6-gRNA01	AtU6-gRNA02	AtUBQ10-zCas9	Hygromycin
pLR12	*PyTFL1.1/1.2*	AtU3-gRNA01	AtU3-gRNA02	AtUBQ10-zCas9	Hygromycin
pLR13	*PyTFL1.1/1.2*	AtU6-gRNA01	AtU6-gRNA03	AtUBQ10-zCas9	Hygromycin
pLR14	*PyTFL1.1/1.2*	AtU3-gRNA01	AtU3-gRNA03	AtUBQ10-zCas9	Hygromycin
pLR15	*PyTFL1.1/1.2*	AtU6-gRNA02	AtU6-gRNA04	AtUBQ10-zCas9	Hygromycin
pLR16	*PyTFL1.1/1.2*	AtU3-gRNA02	AtU3-gRNA04	AtUBQ10-zCas9	Hygromycin
pLR17	*PyPDS*	AtU6-gRNA01	AtU3-gRNA03	35S-zCas9	Kanamycin
pLR18	*PyPDS*	AtU3-gRNA01	AtU6-gRNA03	35S-zCas9	Kanamycin
pLR19	*PyPDS*	AtU6-gRNA02	AtU3-gRNA04	35S-zCas9	Kanamycin
pLR20	*PyPDS*	AtU3-gRNA02	AtU6-gRNA04	35S-zCas9	Kanamycin
pLR21	*PyPDS*	AtU6-gRNA05	AtU3-gRNA06	35S-zCas9	Kanamycin
pLR22	*PyPDS*	AtU3-gRNA05	AtU6-gRNA06	35S-zCas9	Kanamycin
pLR23	*PyGID1*	AtU6-gRNA02	AtU3-gRNA04	35S-zCas9	Kanamycin
pLR24	*PyGID1*	AtU3-gRNA02	AtU6-gRNA04	35S-zCas9	Kanamycin
pLR25	*PyGID1*	AtU6-gRNA01	AtU3-gRNA05	35S-zCas9	Kanamycin
pLR26	*PyGID1*	AtU3-gRNA01	AtU6-gRNA05	35S-zCas9	Kanamycin

Figure S5. PCR identification of the regenerated calli (pLR01-16) using vector-specific primers. M, DNA marker. Plasmid, positive control. WT, wide type. H_2O, negative control.

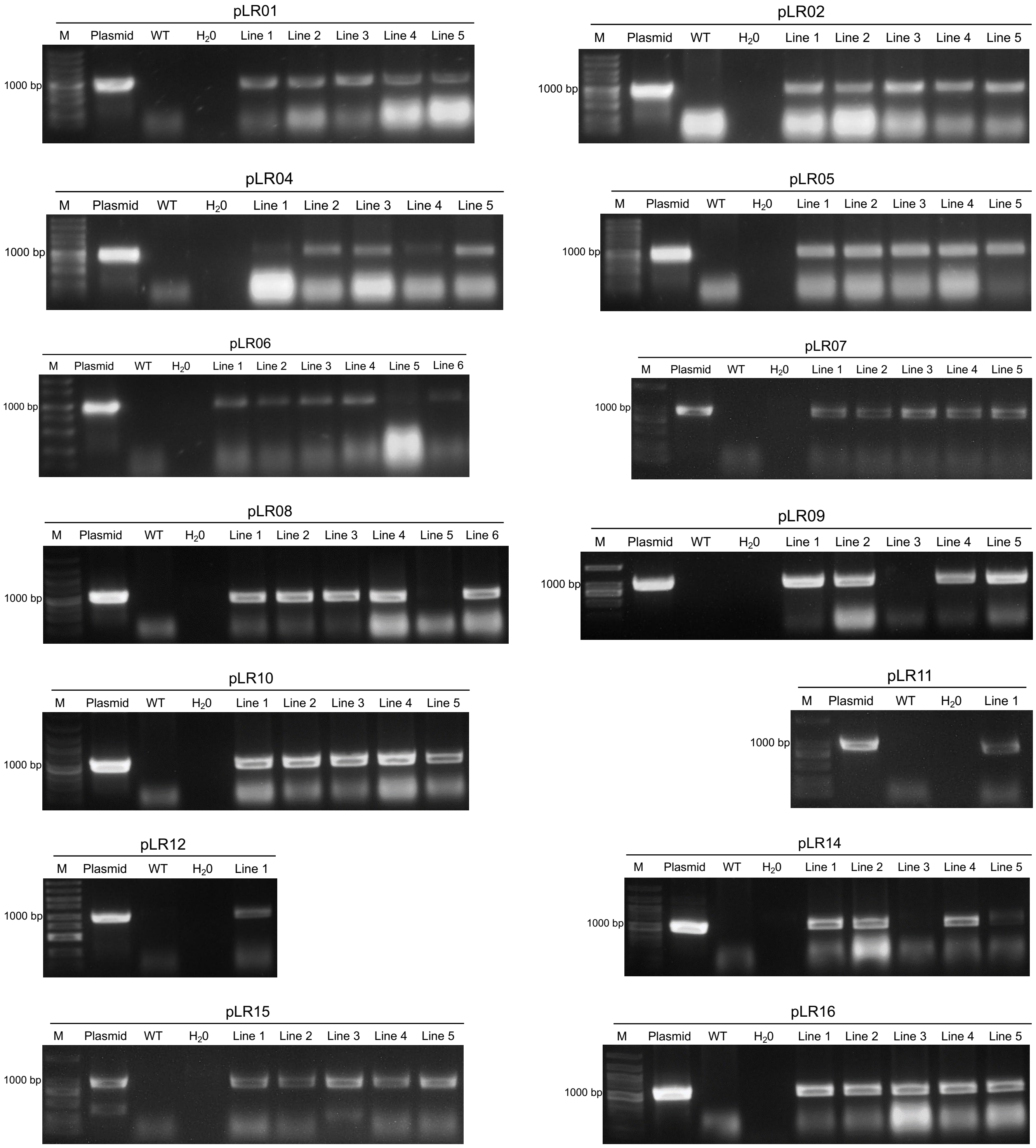

Figure S6. PCR identification of the regenerated calli (pLR55-64) using vector-specific primers. A, M, DNA marker. Plasmid, positive control. WT, wide type. H_2O, negative control. B, Reconstructed vectors with hygromycin gene replaced with kanamycin gene.

A

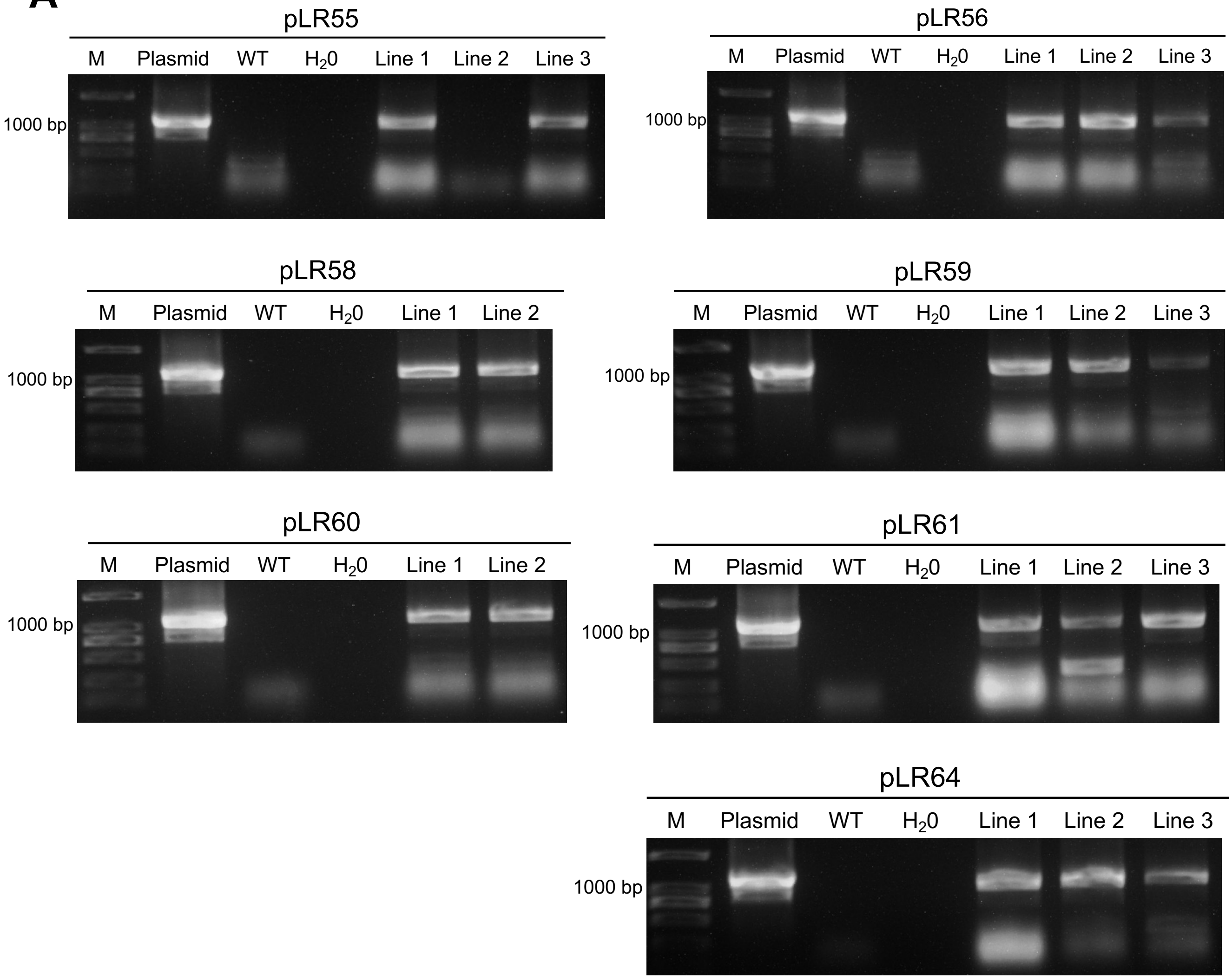

B

Kan					Hyg
**Vectors**	**Target genes**	**sgRNA**		**Cas9**	**Vectors**
pLR17	*PyPDS*	AtU6-gRNA01	AtU3-gRNA03	35S-zCas9	pLR55
pLR18	*PyPDS*	AtU3-gRNA01	AtU6-gRNA03	35S-zCas9	pLR56
pLR19	*PyPDS*	AtU6-gRNA02	AtU3-gRNA04	35S-zCas9	pLR57
pLR20	*PyPDS*	AtU3-gRNA02	AtU6-gRNA04	35S-zCas9	pLR58
pLR21	*PyPDS*	AtU6-gRNA05	AtU3-gRNA06	35S-zCas9	pLR59
pLR22	*PyPDS*	AtU3-gRNA05	AtU6-gRNA06	35S-zCas9	pLR60
pLR23	*PyGID1*	AtU6-gRNA02	AtU3-gRNA04	35S-zCas9	pLR61
pLR24	*PyGID1*	AtU3-gRNA02	AtU6-gRNA04	35S-zCas9	pLR62
pLR25	*PyGID1*	AtU6-gRNA01	AtU3-gRNA05	35S-zCas9	pLR63
pLR26	*PyGID1*	AtU3-gRNA01	AtU6-gRNA05	35S-zCas9	pLR64

Figure S7. Mutation frequencies at *PyPDS*-gRNA05 site by PCR-RFLP and Sanger sequencing analysis. A, PCR-RFLP analysis of CRISPR/Cas9-induced mutation at the *PyPDS*-gRNA05 site. The PflmI enzyme site used for PCR-RFLP analysis is highlighted in green. Mutation frequencies were calculated by Image J. B, Sanger sequencing analysis CRISPR/Cas9-induced mutation at the *PyPDS*-gRNA05 site. Dash indicates 1 bp deletion. Green DNA bases indicate insertion. The PAM sequence is highlighted in red, and the target sequence is highlighted in blue. WT, wild-type sequence.

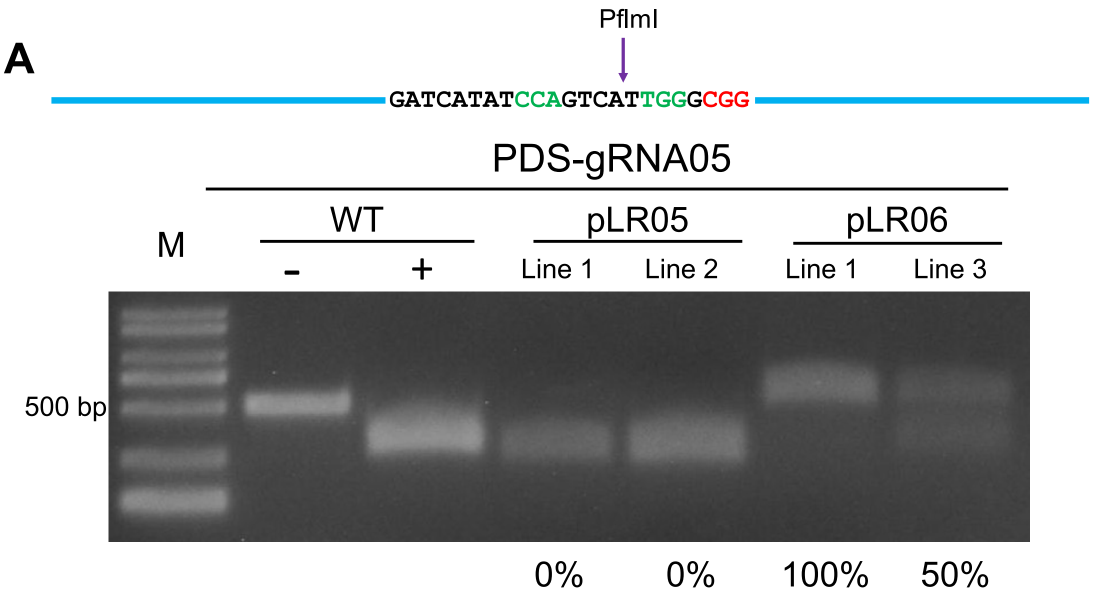

B

```
             PyPDS-gRNA05   pLR06      (10/10) 100%
WT          TCCAATTGTTGATCATATCCAGTCATTGGGCGGTGAAGTCCGAAC
Line1-1     TCCAATTGTTGATCATATCCAGTCATTTGGGCGGTGAAGTCCGAAC   +1bp
Line1-2     TCCAATTGTTGATCATATCCAGTCAT-GGGCGGTGAAGTCCGAAC    -1bp
Line1-3     TCCAATTGTTGATCATATCCAGTCATTTGGGCGGTGAAGTCCGAAC   +1bp
Line1-4     TCCAATTGTTGATCATATCCAGTCATTTGGGCGGTGAAGTCCGAAC   +1bp
Line1-5     TCCAATTGTTGATCATATCCAGTCATTTGGGCGGTGAAGTCCGAAC   +1bp
Line1-6     TCCAATTGTTGATCATA----------GGGCGGTGAAGTCCGAAC    -10bp
Line1-7     TCCAATTGTTGATCATATCCAGTCAT-GGGCGGTGAAGTCCGAAC    -1bp
Line1-8     TCCAATTGTTGATCATATCCAGTCATTTGGGCGGTGAAGTCCGAAC   +1bp
Line1-9     TCCAATTGTTGATCATATCCAGTCAT---------AAGTCCGAAC    -9bp
Line1-10    TCCAATTGTTGATCATATCCAGTCATTTGGGCGGTGAAGTCCGAAC   +1bp

             PyPDS-gRNA05   pLR06      (5/10) 50%
WT          TCCAATTGTTGATCATATCCAGTCATTGGGCGGTGAAGTCCGAAC
Line3-1     TCCAATTGTTGATCATATCCAGTCATTGGGCGGTGAAGTCCGAAC
Line3-2     TCCAATTGTTGATCATATCCAGTCATTTGGGCGGTGAAGTCCGAAC   +1bp
Line3-3     TCCAATTGTTGATCATATCCAGTCAT-GGGCGGTGAAGTCCGAAC    -1bp
Line3-4     TCCAATTGTTGATCATATCCAGTCATTGGGCGGTGAAGTCCGAAC
Line3-5     TCCAATTGTTGATCATATCCAGTCATTGGGCGGTGAAGTCCGAAC
Line3-6     TCCAATTGTTGATCATATCCAGTCAT-GGGCGGTGAAGTCCGAAC    -1bp
Line3-7     TCCAATTGTTGATCATATCCAGTCAT-GGGCGGTGAAGTCCGAAC    -1bp
Line3-8     TCCAATTGTTGATCATATCCAGTCATTTGGGCGGTGAAGTCCGAAC   +1bp
Line3-9     TCCAATTGTTGATCATATCCAGTCATTGGGCGGTGAAGTCCGAAC
Line3-10    TCCAATTGTTGATCATATCCAGTCATTGGGCGGTGAAGTCCGAAC
```

Figure S8. The deletion and insertion frequency of CRISPR/Cas9 system with 35S and AtU3 promoters at target sites of *PyPDS* and *PyGID1*. A, *PyPDS*. B, *PyGID1*.

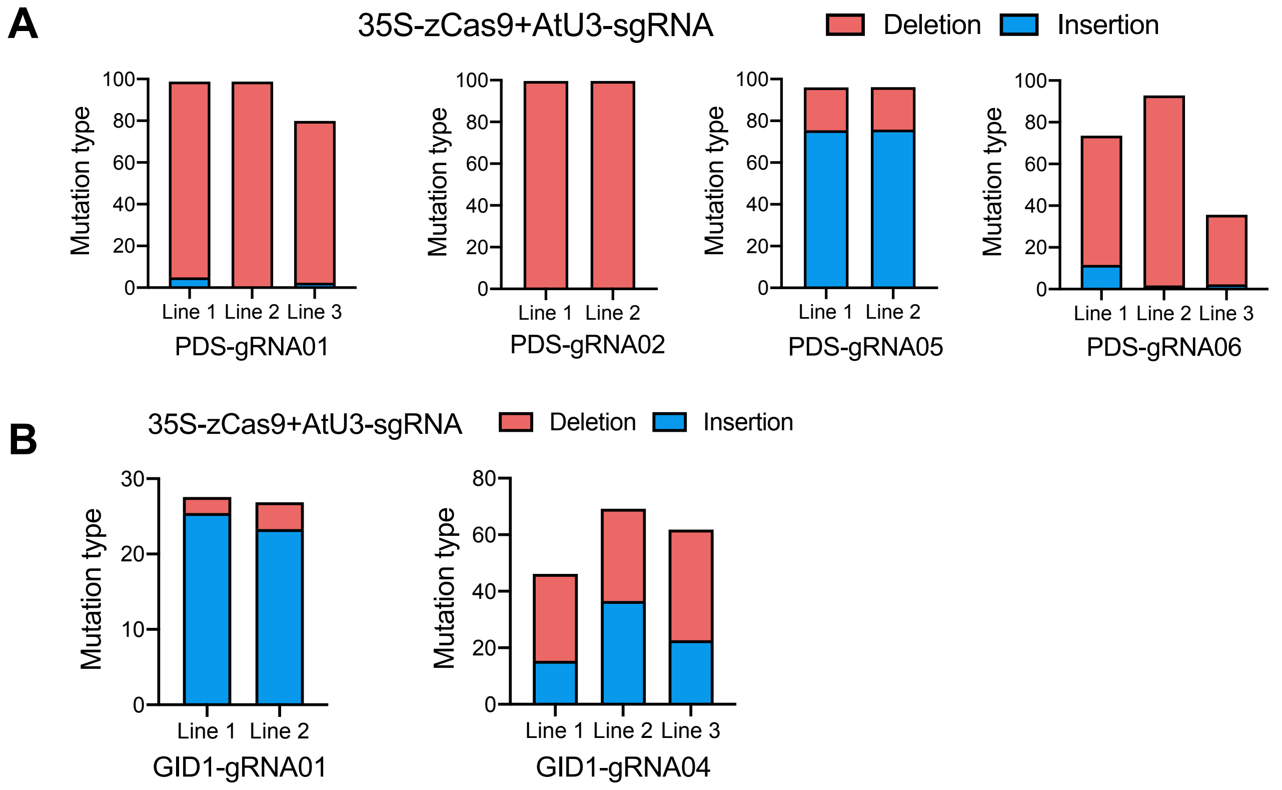

Figure S9. DNA sequence alignment of *PyMYB10* and *PyMYB114.*
REF, reference. CALLUS, dedifferentiated 'Clapp's Favorite' pear calli.

A

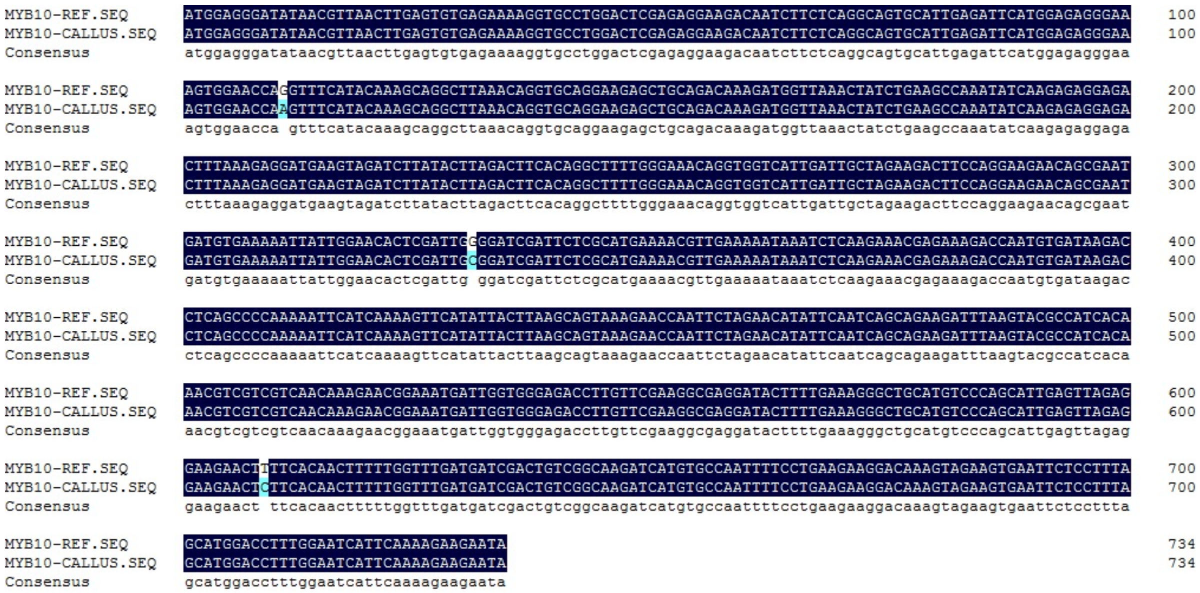

B

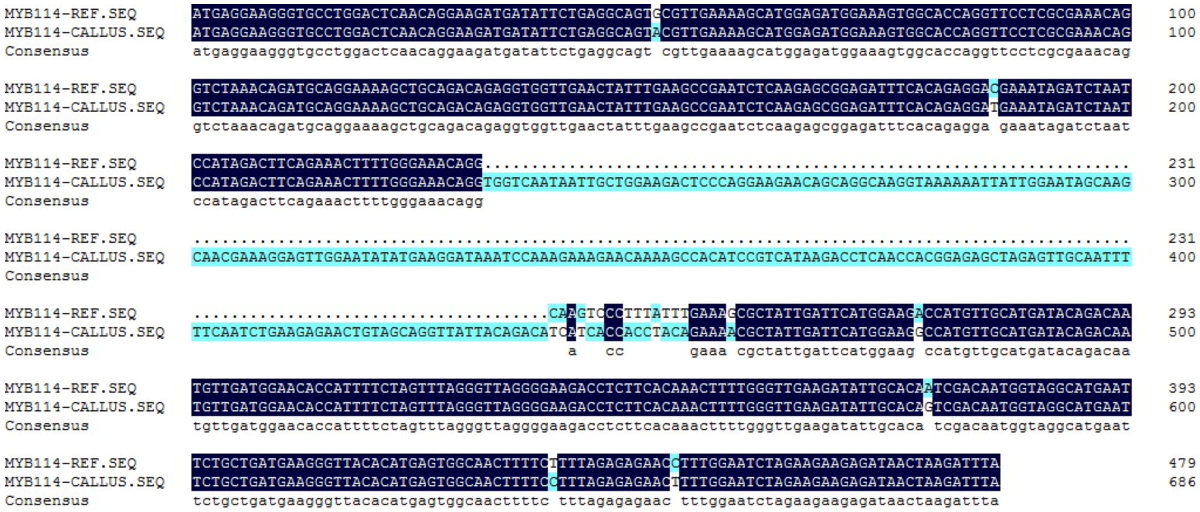

Figure S10. Guide RNA design and construction of CRISPR/Cas12a and Cas12b vectors for genome editing. A, Schematics of the crRNA and sgRNA positions and sequences for CRISPR/Cas12a and Cas12b systems. For Cas12a, 23 nt protospacers with a TTTV PAM are designed. For Cas12 b, 20 nt protospacers with a VTTV PAM are designed. B, Vector constructions of CRISPR/Cas12a and Cas12b systems.

A

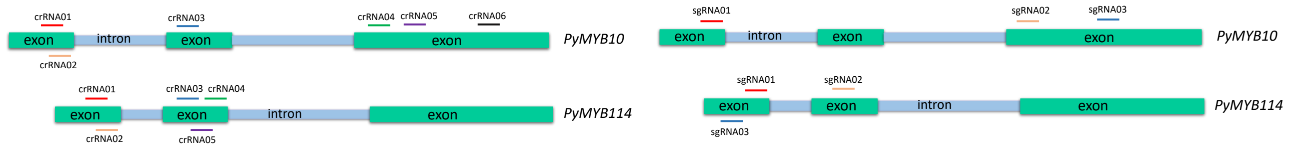

Mb2/LbCas12a [PAM: TTTV + crRNA: 23nt]　　AaCas12b [PAM: VTTV + sgRNA: 20nt]

Target genes	crRNAs	Target sequences	PAM
PyMYB10	crRNA01	CCTCTCCATGAATCTCAATGCAC	TTTC
	crRNA02	TATGAAACTTGGTTCCACTTTCC	TTTG
	crRNA03	ACCATCTTTGTCTGCAGCTCTTC	TTTA
	crRNA04	AACGTTTTCATGCGAGAATCGAT	TTTC
	crRNA05	GGGCTGAGGTCTTATCACATTGG	TTTG
	crRNA06	AAAGGGCTGCATGTCCCAGCATT	TTTG
PyMYB114	crRNA01	AACGTACTGCCTCAGAATATCAT	TTTC
	crRNA02	GCGAGGAACCTGGTGCCACTTTC	TTTC
	crRNA03	AAGCCGAATCTCAAGAGCGGAGA	TTTG
	crRNA04	ACAGAGGATGAAATAGATCTAAT	TTTC
	crRNA05	ATCCTCTGTGAAATCTCCGCTCT	TTTC
Target genes	sgRNAs	Target sequences	PAM
PyMYB10	sgRNA01	AGATTCATGGAGAGGGAAAG	ATTG
	sgRNA02	CTAGAAGACTTCCAGGAAGA	ATTG
	sgRNA03	CTTAAGCAGTAAAGAACCAA	ATTA
PyMYB114	sgRNA01	AAAAGCATGGAGATGGAAAG	GTTG
	sgRNA02	AACTATTTGAAGCCGAATCT	GTTG
	sgRNA03	TGAGGCAGTACGTTGAAAAG	ATTC

B

Vectors	Target genes	Target genes	Cas12
pLR67	*PyMYB10*-crRNA01+crRNA04		Mb2Cas12a
pLR68	*PyMYB10*-crRNA02+crRNA05		Mb2Cas12a
pLR69	*PyMYB10*-crRNA03+crRNA06		Mb2Cas12a
pLR70	*PyMYB114*-crRNA01+crRNA03		Mb2Cas12a
pLR71	*PyMYB114*-crRNA02+crRNA04		Mb2Cas12a
pLR72	*PyMYB114*-crRNA01+crRNA05		Mb2Cas12a
pLR73	*PyMYB10*-crRNA01	*PyMYB114*-crRNA01	Mb2Cas12a
pLR74	*PyMYB10*-crRNA02	*PyMYB114*-crRNA02	Mb2Cas12a
pLR75	*PyMYB10*-crRNA03	*PyMYB114*-crRNA03	Mb2Cas12a
pLR78	*PyMYB10*-crRNA01+crRNA04	*PyMYB114*-crRNA01+crRNA03	LbCas12a
pLR79	*PyMYB10*-crRNA02+crRNA05	*PyMYB114*-crRNA02+crRNA04	LbCas12a
pLR80	*PyMYB10*-crRNA03+crRNA06	*PyMYB114*-crRNA01+crRNA05	LbCas12a
pLR81	*PyMYB10*-sgRNA01	*PyMYB114*-sgRNA01	AaCas12b
pLR82	*PyMYB10*-sgRNA02	*PyMYB114*-sgRNA02	AaCas12b
pLR83	*PyMYB10*-sgRNA03	*PyMYB114*-sgRNA03	AaCas12b

Figure S11. PCR identification of the regenerated calli (pLR67-83) using vector-specific primers. M, DNA marker. Plasmid, positive control. WT, wide type. H_2O, negative control.

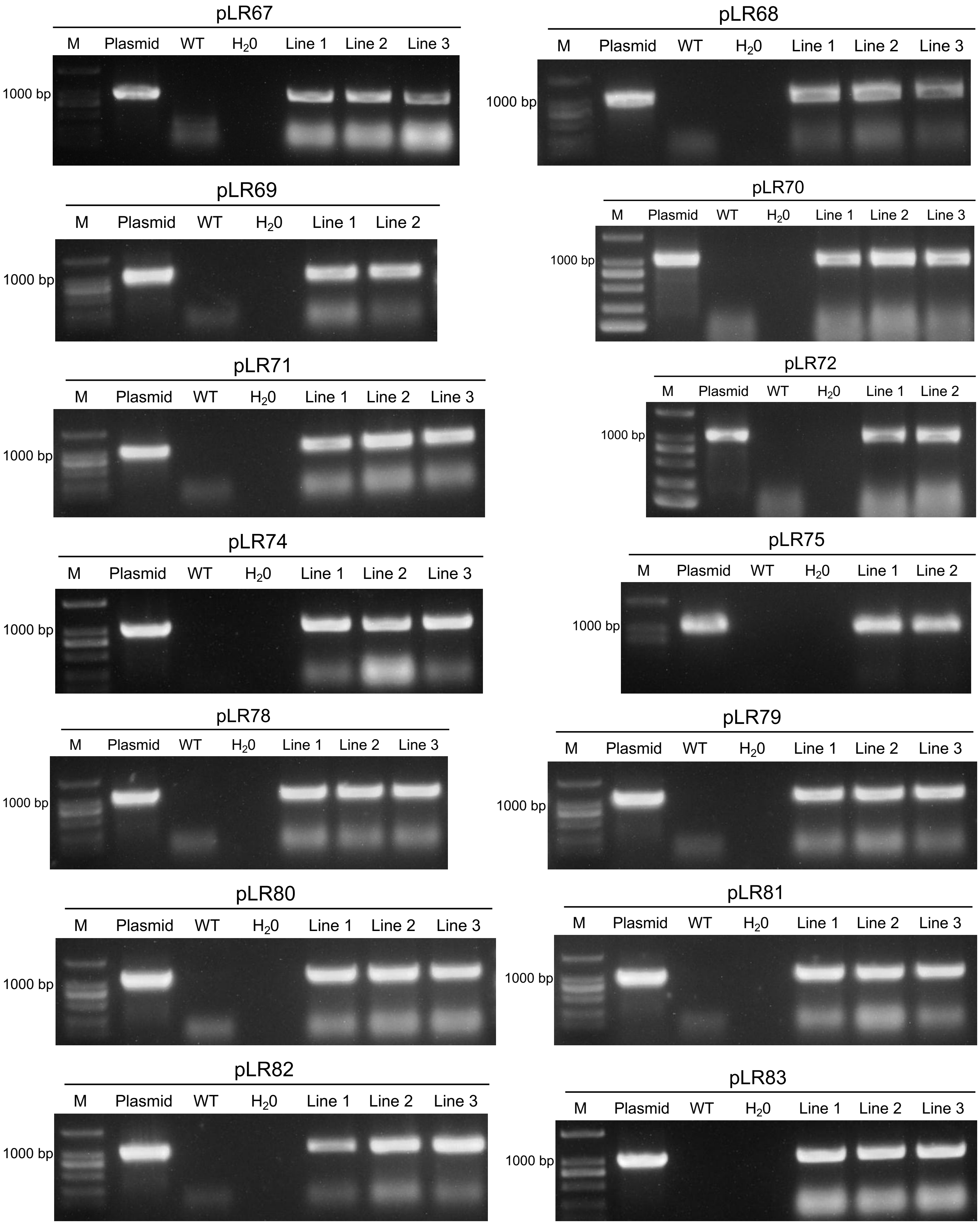

Figure S12. DNA sequence alignment of *PyMYB169* and *PyNSC.* REF, reference. DS, ‘Dangshansuli’ pear fruits. CALLUS, dedifferentiated ‘Clapp’s Favorite’ pear calli.

A

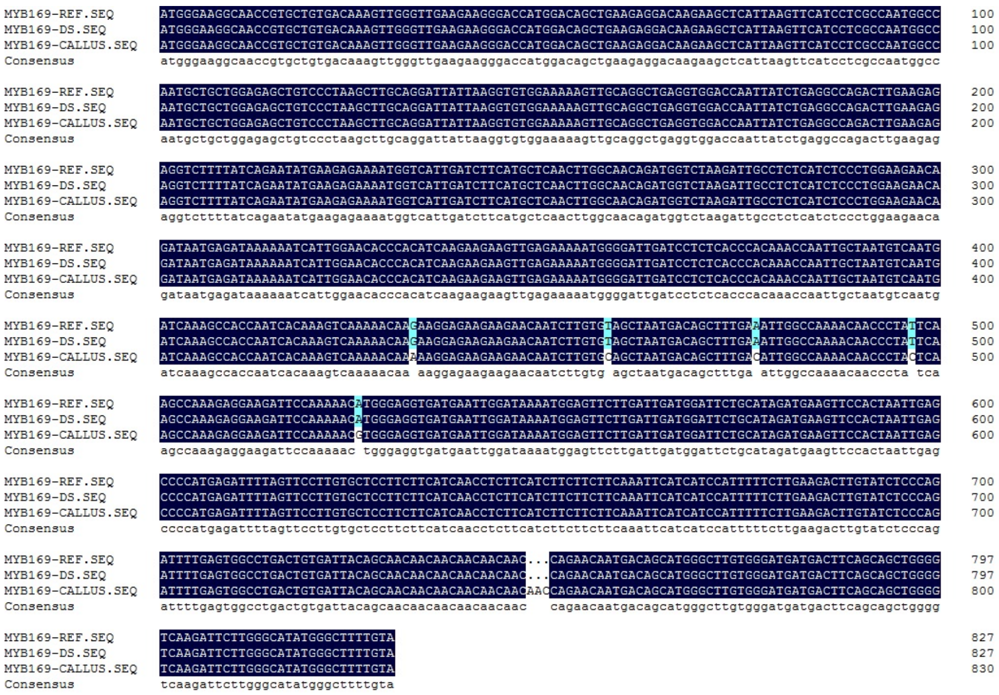

B

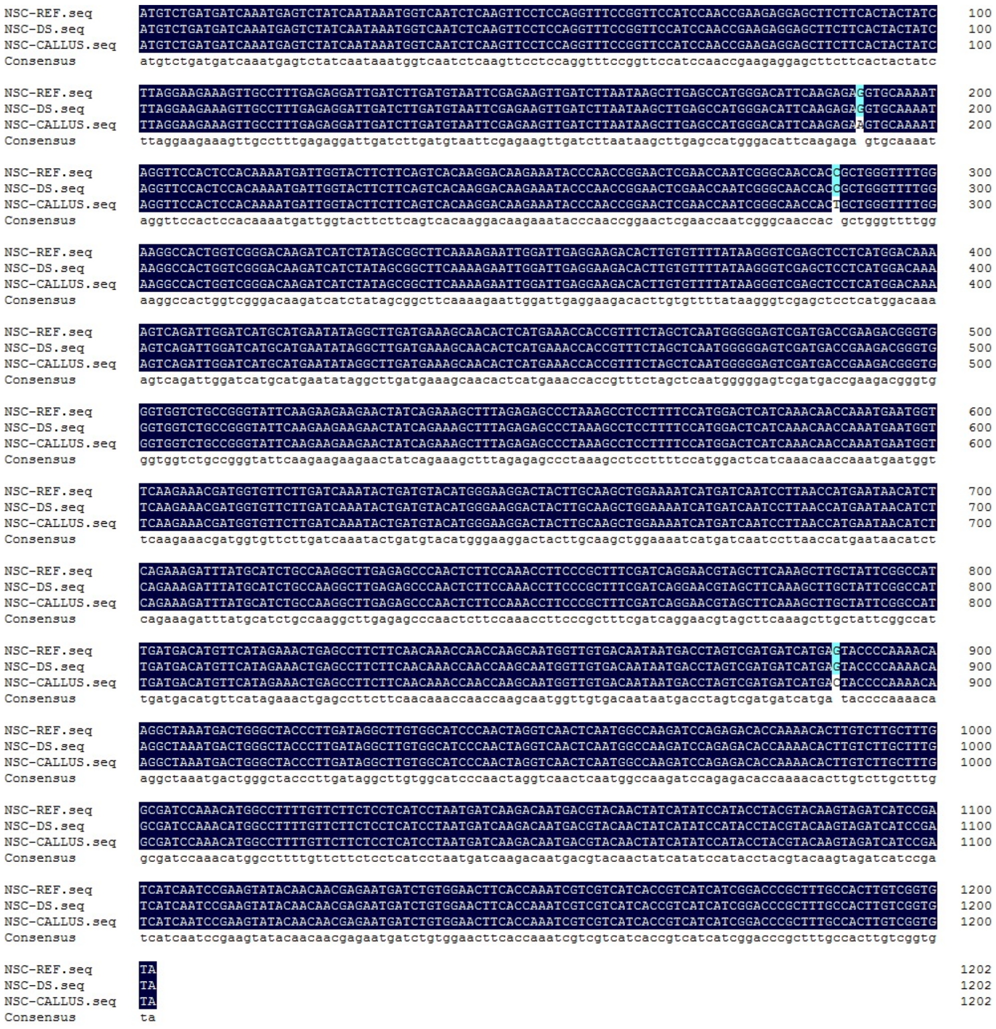

Figure S13. Guide RNA design for anthocyanin and lignin biosynthetic genes knockout by CRISPR/Cas9. A, Schematics of the sgRNA positions and sequences of *PyMYB10, PyMYB114, PyMYB169* and *PyNSC* for genome editing. For Cas9, 20 nt protospacers with a NGG PAM are designed. B, Vector construction of CRISPR/Cas9 system for anthocyanin and lignin engineering.

A

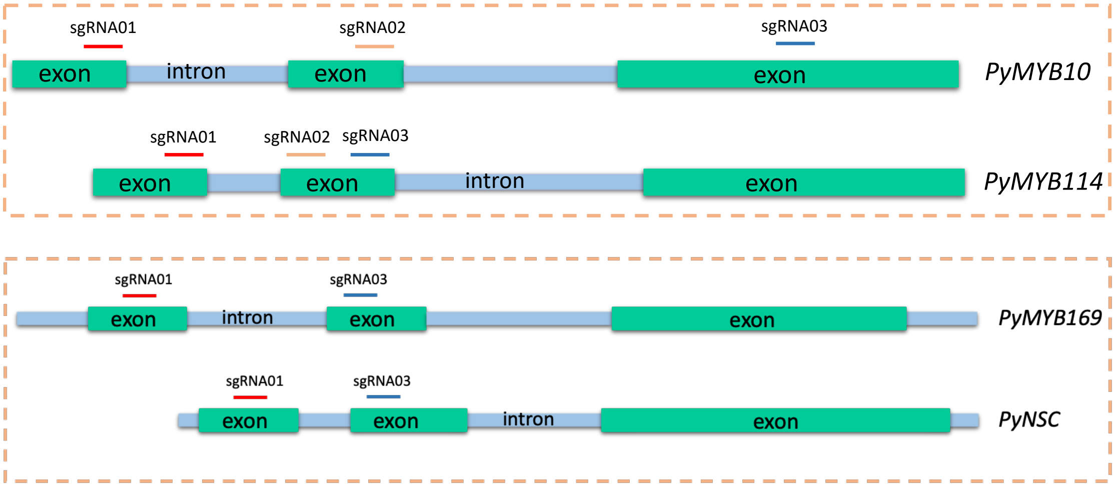

Target genes	sgRNAs	Target sequences	PAM
PyMYB10	sgRNA01	AACCAAGTTTCATACAAAGC	AGG
	sgRNA02	ACTTAGACTTCACAGGCTTT	TGG
	sgRNA03	ACGTCGTCGTCAACAAAGAA	CGG
PyMYB114	sgRNA01	AAAAGCATGGAGATGGAAAG	TGG
	sgRNA02	GCAGGAAAAGCTGCAGACAG	AGG
	sgRNA03	CAAGAGCGGAGATTTCACAG	AGG
PyMYB169	sgRNA01	TTGTCCTCTTCAGCTGTCCA	TGG
	sgRNA03	TGAGGTGGACCAATTATCTG	AGG
PyNSC	sgRNA01	CCGGTTCCATCCAACCGAAG	AGG
	sgRNA03	ACCAATCGGGCAACCACCGC	TGG

zCas9 [sgRNA: 20nt + PAM: NGG]

B

**Vectors**	**Target genes**	**Target genes**	**Cas9**
pLR89	*PyMYB10*-gRNA01	*PyMYB114*-gRNA01	AtUBQ10-zCas9
pLR90	*PyMYB10*-gRNA02	*PyMYB114*-gRNA02	AtUBQ10-zCas9
pLR91	*PyMYB10*-gRNA03	*PyMYB114*-gRNA03	AtUBQ10-zCas9
pLR92	*PyMYB169*-gRNA01+gRNA03		AtUBQ10-zCas9
pLR94	*PyNSC*-gRNA01+gRNA03		AtUBQ10-zCas9

Figure S14. PCR identification of the regenerated calli (pLR89-94) using vector-specific primers. M, DNA marker. Plasmid, positive control. WT, wide type. H_2O, negative control.

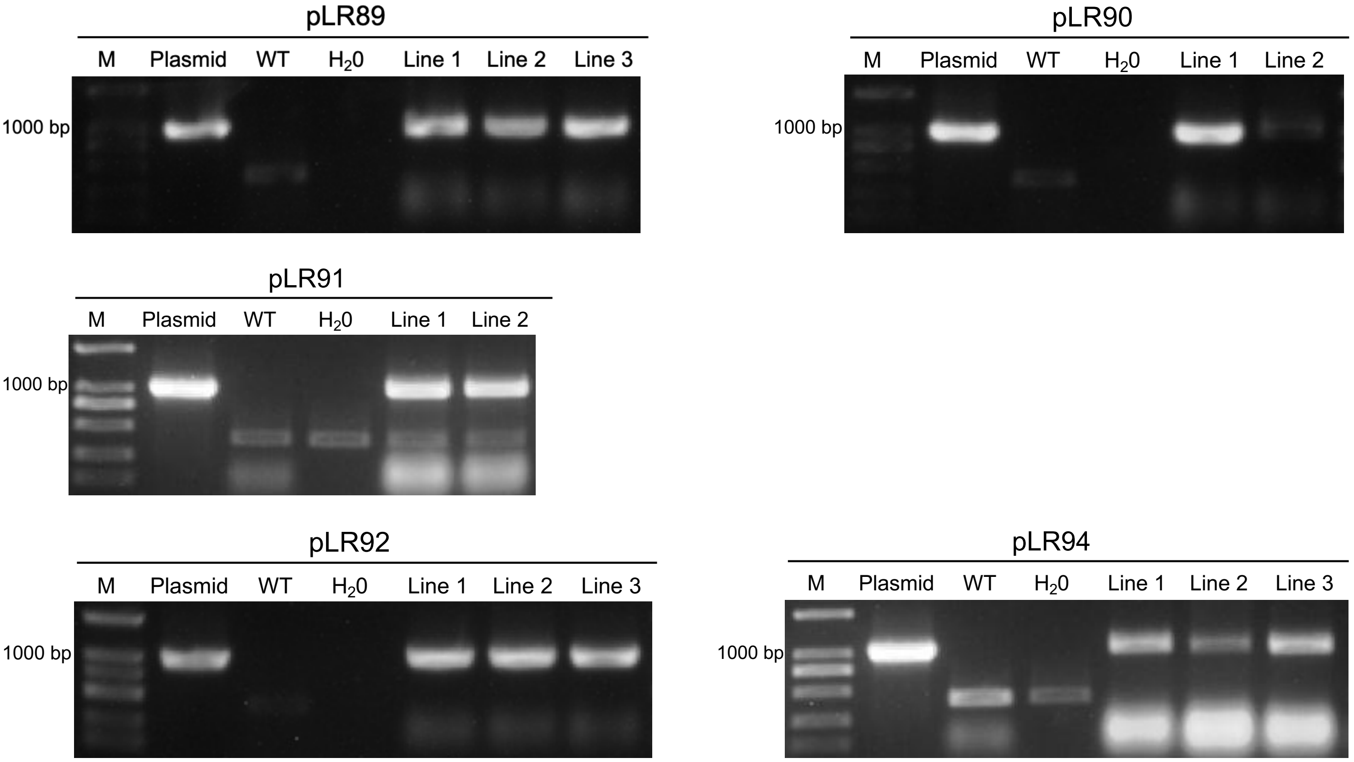

Figure S15. Sanger sequencing results (pLR89-91). The number represent mutated clones / sequenced clones. Dash indicates 1 bp deletion. Green DNA bases indicate insertion. The PAM sequence is highlighted in red, and the target sequence is highlighted in blue. WT, wild-type sequence.

PyMYB10-gRNA01 pLR89 (10/10) 100%

```
WT         GGGAAAGTGGAACCAAGTTTCATACAAAGCAGGTATATATGTTAA
Line1-1    GGGAAAGTGGAACCAAGTTTCATACAAAAGCAGGTATATATGTTAA   +1bp
Line1-2    GGGAAAGTGGAACCAAGTTTCATACAA-GCAGGTATATATGTTAA    -1bp
Line1-3    GGGAAAGTGGAACCAAGTTTCATACAAAAGCAGGTATATATGTTAA   +1bp
Line1-4    GGGAAAGTGGAACCAAGTTTCATAC--------TATATATGTTAA    -8bp
Line1-5    GGGAAAGTGGAACCAAGTTTCATACAAGAGCAGGTATATATGTTAA   +1bp
Line1-6    GGGAAAGTGGAACCAAGTTTCATACAAAAGCAGGTATATATGTTAA   +1bp
Line1-7    GGGAAAGTGGAACCAAGTTTCATACAAAAGCAGGTATATATGTTAA   +1bp
Line1-8    GGGAAAGTGGAACCAAGTTTCATACAAGAGCAGGTATATATGTTAA   +1bp
Line1-9    GGGAAAGTGGAACCAAGTTTCATACAAGAGCAGGTATATATGTTAA   +1bp
Line1-10   GGGAAAGTGGAACCAAGTTTCATACAAGAGCAGGTATATATGTTAA   +1bp
```

PyMYB10-gRNA01 pLR89 (7/8) 87.5%

```
WT         GGGAAAGTGGAACCAAGTTTCATACAAAGCAGGTATATATGTTAA
Line2-1    GGGAAAGTGGAACCAAGTTTCATACAA-GCAGGTATATATGTTAA    -1bp
Line2-2    GGGAAAGTGGAACCAAGTTTCATACAA-GCAGGTATATATGTTAA    -1bp
Line2-3    GGGAAAGTGGAACCAAGTTTCATACAA------------------    -24bp
Line2-4    GGGAAAGTGGAACCAAGTTTCATACAA------------------    -24bp
Line2-5    GGGAAAGTGGAACCAAGTTTCATACAA-GCAGGTATATATGTTAA    -1bp
Line2-6    GGGAAAGTGGAACCAAGTTTCATA----GCAGGTATATATGTTAA    -4bp
Line2-7    GGGAAAGTGGAACCAAGTTTCATACAAAGCAGGTATATATGTTAA
Line2-8    GGGAAAGTGGAACCAAGTTTCATACAAAAGCAGGTATATATGTTAA   +1bp
```

PyMYB10-gRNA01 pLR89 (5/7) 71.4%

```
WT         GGGAAAGTGGAACCAAGTTTCATACAAAGCAGGTATATATGTTAA
Line3-1    GGGAAAGTGGAACCAAGTTTCATACAAAAGCAGGTATATATGTTAA   +1bp
Line3-2    GGGAAAGTGGAACCAAGTTTCATACAA-GCAGGTATATATGTTAA    -1bp
Line3-3    GGGAAAGTGGAACCAAGTTTCATACAAAAGCAGGTATATATGTTAA   +1bp
Line3-4    GGGAAAGTGGAACCAAGTTTCATACAAAGCAGGTATATATGTTAA
Line3-5    GGGAAAGTGGAACCAAGTTTCATACAA-GCAGGTATATATGTTAA    -1bp
Line3-6    GGGAAAGTGGAACCAAGTTTCATACAA-GCAGGTATATATGTTAA    -1bp
Line3-7    GGGAAAGTGGAACCAAGTTTCATACAAAGCAGGTATATATGTTAA
```

PyMYB10-gRNA02 pLR90 (5/10) 50%

```
WT         TAGATCTTATACTTAGACTTCACAGGCTTTTGGGAAACAGGTACT
Line1-1    TAGATCTTATACTTAGACTTCACAGGCTTTTGGGAAACAGGTACT
Line1-2    TAGATCTTATACTTAGACTTCACAGGCTTTTGGGAAACAGGTACT
Line1-3    TAGATCTTATACTTAGACTTCACAGG-------GAAACAGGTACT    -7bp
Line1-4    TAGATCTTATACTTAGACTTCACAGGCTTTTGGGAAACAGGTACT
Line1-5    TAGATCTTATACTTAGACTTCACAGGCATTTTGGGAAACAGGTACT   +1bp
Line1-6    TAGATCTTATACTTAGACTTCACAGGCTTTTGGGAAACAGGTACT
Line1-7    TAGATCTTATACTTAGACTTCACAGGCTTTTGGGAAACAGGTACT
Line1-8    TAGATCTTATACTTAGACTTCACAGGCTTTTTGGGAAACAGGTACT   +1bp
Line1-9    TAGATCTTATACTTAGACTTCACAGGCATTTTGGGAAACAGGTACT   +1bp
Line1-10   TAGATCTTATACTTAGACTTCACAGG---------------TACT     -15bp
```

PyMYB10-gRNA02 pLR90 (5/10) 50%

```
WT         TAGATCTTATACTTAGACTTCACAGGCTTTTGGGAAACAGGTACT
Line2-1    TAGATCTTATACTTAGACTTCACAGGCTTTTTGGGAAACAGGTACT   +1bp
Line2-2    TAGATCTTATACTTAGACTTCACAGGCTTTTGGGAAACAGGTACT
Line2-3    TAGATCTTATACTTAGACTTCACAGG-TTTTGGGAAACAGGTACT    -1bp
Line2-4    TAGATCTTATACTTAGACTTCACAGGCTTTTGGGAAACAGGTACT
Line2-5    TAGATCTTATACTTAGACTTCACAGGCATTTTGGGAAACAGGTACT   +1bp
Line2-6    TAGATCTTATACTTAGACTTCACAGGCTTTTGGGAAACAGGTACT
Line2-7    TAGATCTTATACTTAGACTTCACAGGCTTTTGGGAAACAGGTACT
Line2-8    TAGATCTTATACTTAGACTTCACAGGCTTTTTGGGAAACAGGTACT   +1bp
Line2-9    TAGATCTTATACTTAGACTTCACAGGCATTTTGGGAAACAGGTACT   +1bp
Line2-10   TAGATCTTATACTTAGACTTCACAGGCTTTTGGGAAACAGGTACT
```

PyMYB10-gRNA03 pLR91 (10/10) 100%

```
WT         GCCATCACAAACGTCGTCGTCAACAAAGAACGGAAATGATTGGTG
Line2-1    GCCATCACAAACGTCGTCGTCAACAAAAGAACGGAAATGATTGGTG   +1bp
Line2-2    GCCATCACAAACGT--------------AACGGAAATGATTGGTG    -14bp
Line2-3    GCCATCACAAACGCCTTC---------GAACGGAAATGATTGGTG    -14bp,+5bp
Line2-4    --------------------------AGAACGGAAATGATTGGTG    -116bp
Line2-5    ---------------------------------------------    -78bp,+28bp
Line2-6    GCCATCACAAACGTCGTCGTCAACAAAAGAACGGAAATGATTGGTG   +1bp
Line2-7    GCCATCACAAACGTCGTCGTCAACAAAAGAACGGAAATGATTGGTG   +1bp
Line2-8    GCCATCACAAACGTCGTCGTCAACAAAAGAACGGAAATGATTGGTG   +1bp
Line2-9    GCCATCACAAACGTCGTCGTCAAC---GAACGGAAATGATTGGTG    -3bp
Line2-10   GCCATCACAAACGTCGTCGTCAA----GAACGGAAATGATTGGTG    -4bp
```

PyMYB114-gRNA01 pLR89 (9/10) 90%

```
WT         CAGTACGTTGAAAAGCATGGAGATGGAAAGTGGCACCAGGTTCCT
Line1-1    CAGTACGTTGAAAAGCATGGAGAT--AAAGTGGCACCAGGTTCCT    -2bp
Line1-2    CAGTACGTTGAAAAGCATGGAGATGGAAAAGTGGCACCAGGTTCCT   +1bp
Line1-3    CAGTACGTTGAAAAGCATGGAGAT---AAGTGGCACCAGGTTCCT    -3bp
Line1-4    CAGTACGTTGAAAAGCATGGAGATGGAAAGTGGCACCAGGTTCCT
Line1-5    CAGTACGTTGAAAAGCATGGAGATGGAAAAGTGGCACCAGGTTCCT   +1bp
Line1-6    CAGTACGTTGAAAAGCATGGAGATGGA-----GCACCAGGTTCCT    -5bp
Line1-7    CAGTACGTTGAAAAGCATGGAGATGG---GTGGCACCAGGTTCCT    -3bp
Line1-8    CAGTACGTTGAAAAGCATGGAGAT--AAAGTGGCACCAGGTTCCT    -2bp
Line1-9    CAGTACGTTGAAAAGCATGGAGAT--AAAGTGGCACCAGGTTCCT    -2bp
Line1-10   ------------------------------------CAGGTTCCT    -42bp
```

PyMYB114-gRNA01 pLR89 (4/10) 40%

```
WT         CAGTACGTTGAAAAGCATGGAGATGGAAAGTGGCACCAGGTTCCT
Line2-1    CAGTACGTTGAAAAGCATGGAGATGGAAAGTGGCACCAGGTTCCT
Line2-2    CAGTACGTTGAAAAGCATGGAGATGGAAAGTGGCACCAGGTTCCT
Line2-3    CAGTACGTTGAAAAGCATGGAGATGGAAAGTGGCACCAGGTTCCT
Line2-4    CAGTACGTTGAAAAGCATGGAGATGGAAAAGTGGCACCAGGTTCCT   +1bp
Line2-5    ---------------------------------------------    -53bp
Line2-6    CAGTACGTTGAAAAGCATGGAGATGGAAAGTGGCACCAGGTTCCT
Line2-7    CAGTACGTTGAAAAGCA-----------AGTGGCACCAGGTTCCT    -11bp
Line2-8    CAGTACGTTGAAAAGCATGGAGATGGAAAAGTGGCACCAGGTTCCT   +1bp
Line2-9    CAGTACGTTGAAAAGCATGGAGATGGAAAGTGGCACCAGGTTCCT
Line2-10   CAGTACGTTGAAAAGCATGGAGATGGAAAGTGGCACCAGGTTCCT
```

PyMYB114-gRNA01 pLR89 (7/10) 70%

```
WT         CAGTACGTTGAAAAGCATGGAGATGGAAAGTGGCACCAGGTTCCT
Line3-1    CAGTACGTTGAAAAGCATGGAGATGGAAAGTGGCACCAGGTTCCT
Line3-2    CAGTACGTTGAAAAGCATGGAGATGGAAAGTGGCACCAGGTTCCT
Line3-3    CAGTACGTTGAAAAGCATGGAGAT--AAAGTGGCACCAGGTTCCT    -2bp
Line3-4    CAGTACGTTGAAAAGCATGGAGATGGAAAAGTGGCACCAGGTTCCT   +1bp
Line3-5    CAGTACGTTGAAAAGCATGGAGATGGAAAGTGGCACCAGGTTCCT
Line3-6    CAGTACGTTGAAAAGCATGGAGAT---AAGTGGCACCAGGTTCCT    -3bp
Line3-7    CAGTACGTTGAAAAGCATGGAGA---------------GGTTCCT    -15bp
Line3-8    CAGTACGTTGAAAAGCATGGAGATGGAA-GTGGCACCAGGTTCCT    -1bp
Line3-9    CAGTACGTTGAAAAGCATGGAGATGGAA-GTGGCACCAGGTTCCT    -1bp
Line3-10   CAGTACGTTGAAAAGCATGGAGATGGAAAAGTGGCACCAGGTTCCT   +1bp
```

PyMYB114-gRNA02 pLR90 (10/10) 100%

```
WT         CTAAACAGATGCAGGAAAAGCTGCAGACAGAGGTGGTTGAACTAT
Line1-1    CTAAACAGATGCAGGAAAAGCTGCAGA--GAGGTGGTTGAACTAT    -2bp
Line1-2    CTAAACAGATGCAGGAAAAGCTGCAGAACAGAGGTGGTTGAACTAT   +1bp
Line1-3    CTAAACAGATGCAGGAAAAGCTGCAGAACAGAGGTGGTTGAACTAT   +1bp
Line1-4    CTAAACAGATGCAGGAAAAGCTGCAGA--GAGGTGGTTGAACTAT    -2bp
Line1-5    CTAAACAGATGCAGGAAAAGCTGCAGAACAGAGGTGGTTGAACTAT   +1bp
Line1-6    CTAAACAGATGCAGGAAAAGCTGCAGAACAGAGGTGGTTGAACTAT   +1bp
Line1-7    CTAAACAGATGCAGGAAAAGCTGCAGAACAGAGGTGGTTGAACTAT   +1bp
Line1-8    CTAAACAGATGCAGGAAAAGCTGCAGAACAGAGGTGGTTGAACTAT   +1bp
Line1-9    CTAAACAGATGCAGGAAAAGCTGCAGAA-AG--GTGGTTGAACTAT   +1bp,-3bp
Line1-10   CTAAACAGATGCAGGAAAAGCTGCAGAACAGAGGTGGTTGAACTAT   +1bp
```

PyMYB114-gRNA02 pLR90 (10/10) 100%

```
WT         CTAAACAGATGCAGGAAAAGCTGCAGACAGAGGTGGTTGAACTAT
Line2-1    CTAAACAGATGCAGGAAAAGCTGCAGAACAGAGGTGGTTGAACTAT   +1bp
Line2-2    CTAAACAGATGCAGGAAAAGCTGCAGAACAGAGGTGGTTGAACTAT   +1bp
Line2-3    CTAAACAGATGCAGGAAAAGCTGCAGAACAGAGGTGGTTGAACTAT   +1bp
Line2-4    CTAAACAGATGCAGGAAAAGCTGCAGAACAGAGGTGGTTGAACTAT   +1bp
Line2-5    CTAAACAGATGCAGGAAAAGCTGCAGAACAGAGGTGGTTGAACTAT   +1bp
Line2-6    CTAAACAGATGCAGGAAAAGCTGCAGAACAGAGGTGGTTGAACTAT   +1bp
Line2-7    CTAAACAGATGCAGGAAAAGCTGCAGAACAGAGGTGGTTGAACTAT   +1bp
Line2-8    CTAAACAGATGCAGGAAAAGCTGCAGAACAGAGGTGGTTGAACTAT   +1bp
Line2-9    CTAAACAGATGCAGGAAAAGCTGCAGAACAGAGGTGGTTGAACTAT   +1bp
Line2-10   CTAAACAGATGCAGGAAAAGCTGCAGATCAGAGGTGGTTGAACTAT   +1bp
```

PyMYB114-gRNA03 pLR91 (10/10) 100%

```
WT         AGCCGAATCTCAAGAGCGGAGATTTCACAGAGGATGAAATAGATC
Line2-1    AGCCGAATCTCAAGAGCGGAGATTTCAACAGAGGATGAAATAGATC   +1bp
Line2-2    AGCCGAATCTCAAGAGCGGAGAT---ACAGAGGATGAAATAGATC    -3bp
Line2-3    AGCCGAATCTCAAGAGCGGAGA-----CAGAGGATGAAATAGATC    -5bp
Line2-4    AGCCGAATCTCAAGAGCGGAGATTTCAACAGAGGATGAAATAGATC   +1bp
Line2-5    AGCCGAATCTCAAGAGCGGAGATTTCAACAGAGGATGAAATAGATC   +1bp
Line2-6    AGCCGAATCTCAAGAGCGGAGAT---ACAGAGGATGAAATAGATC    -3bp
Line2-7    AGCCGAATCTCAAGAGCGGAGATTTCAACAGAGGATGAAATAGATC   +1bp
Line2-8    AGCCGAATCTCAAGAGCGGAGATTTCATCAGAGGATGAAATAGATC   +1bp
Line2-9    AGCCGAATCTCAAGAGCGGAGA-----CAGAGGATGAAATAGATC    -5bp
Line2-10   AGCCGAATCTCAAGAGCGGAGATTTCAACAGAGGATGAAATAGATC   +1bp
```

Figure S16. Sanger sequencing results (pLR92, 94). The number represent mutated clones / sequenced clones. Dash indicates 1 bp deletion. Green DNA bases indicate insertion. The PAM sequence is highlighted in red, and the target sequence is highlighted in blue. WT, wild-type sequence.

```
              PyMYB169-gRNA01   pLR92      (1/10)  10%
WT         AATGAGCTTCTTGTCCTCTTCAGCTGTCCATGGTCCCTTCTTCAA
Line1-1    AATGAGCTTCTTGTCCTCTTCAGCTGTCCATGGTCCCTTCTTCAA
Line1-2    AATGAGCTTCTTGTCCTCTTCAGCTGTCCATGGTCCCTTCTTCAA
Line1-3    AATGAGCTTCTTGTCCTCTTCAGCTGTCCATGGTCCCTTCTTCAA
Line1-4    AATGAGCTTCTTGTCCTCTTCAGCTGTCCATGGTCCCTTCTTCAA
Line1-5    AATGAGCTTCTTGTCCTCTTCAGCTGTCCATGGTCCCTTCTTCAA
Line1-6    AATGAGCTTCTTGTCCTCTTCAGCTGTCCATGGTCCCTTCTTCAA
Line1-7    AATGAGCTTCTTGTCCTCTTCAGCTGTCCATGGTCCCTTCTTCAA
Line1-8    AATGAGCTTCTTGTCCTCTTCAGCTGTCCATGGTCCCTTCTTCAA
Line1-9    AATGAGCTTCTTGTCCTCTTCAGCT-TCCATGGTCCCTTCTTCAA   -1bp
Line1-10   AATGAGCTTCTTGTCCTCTTCAGCTGTCCATGGTCCCTTCTTCAA
```

```
              PyMYB169-gRNA01   pLR92      (8/10)  80%
WT         AATGAGCTTCTTGTCCTCTTCAGCTGTCCATGGTCCCTTCTTCAA
Line2-1    AATGAGCTTCTTGTCCTCTTCA-----CCATGGTCCCTTCTTCAA   -5bp
Line2-2    AATGAGCTTCTTGTCCTCTTCAGC--TCCATGGTCCCTTCTTCAA   -2bp
Line2-3    AATGAGCTTCTTGTCCTCTTCAG-----CATGGTCCCTTCTTCAA   -5bp
Line2-4    AATGAGCTTCTTGTCCTCTTCAGCTGTCCATGGTCCCTTCTTCAA
Line2-5    AATGAGCTTCTTGTCCTCTTCAGC--TCCATGGTCCCTTCTTCAA   -2bp
Line2-6    AATGAGCTTCTTGTCCTCTTCAGCTGTCCATGGTCCCTTCTTCAA
Line2-7    AATGAGCTTCTTGTCCTCTTCAGCTGTTCCATGGTCCCTTCTTCAA  +1bp
Line2-8    AATGAGCTTCTTGTCCTCTTCAG-----CATGGTCCCTTCTTCAA   -5bp
Line2-9    AATGAGCTTCTTGTCCTCTTCAGC--TCCATGGTCCCTTCTTCAA   -2bp
Line2-10   AATGAGCTTCTTGTCCTCTTCA-----CCATGGTCCCTTCTTCAA   -5bp
```

```
              PyMYB169-gRNA01   pLR92      (9/10)  90%
WT         AATGAGCTTCTTGTCCTCTTCAGCTGTCCATGGTCCCTTCTTCAA
Line3-1    AATGAGCTTCTTGTCCTCTTCAGCTGTTCCATGGTCCCTTCTTCAA  +1bp
Line3-2    AATGAGCTTCTTGTCCTCTTCAGCTGTTCCATGGTCCCTTCTTCAA  +1bp
Line3-3    AATGAGCTTCTTGTCCTCTTCAGCTGTTCCATGGTCCCTTCTTCAA  +1bp
Line3-4    AATGAGCTTCTTGTCCTCTTCAGCTG-CCATGGTCCCTTCTTCAA   -1bp
Line3-5    AATGAGCTTCTTGTCCTCTTCAGCTG-CCATGGTCCCTTCTTCAA   -1bp
Line3-6    AATGAGCTTCTTGTCCTCTTCA----TCCATGGTCCCTTCTTCAA   -4bp
Line3-7    AATGAGCTTCTTGTCCTCTTCAGCTGTTCCATGGTCCCTTCTTCAA  +1bp
Line3-8    AATGAGCTTCTTGTCCTCTTCAGC--TCCATGGTCCCTTCTTCAA   -2bp
Line3-9    AATGAGCTTCTTGTCCTCTTCAGCTGTCCATGGTCCCTTCTTCAA
Line3-10   AATGAGCTTCTTGTCCTCTTCAGCTGTTCCATGGTCCCTTCTTCAA  +1bp
```

```
              PyNSC-gRNA01   pLR94      (10/10)  100%
WT         CTCCAGGTTTCCGGTTCCATCCAACCGAAGAGGAGCTTCTTCACT
Line1-1    CTCCAGGTTTCCGGTTCCATCCA------GAGGAGCTTCTTCACT   -6bp
Line1-2    CTCCAGGTTTCCGGTTCCATCCAACCGGAAGAGGAGCTTCTTCACT  +1bp
Line1-3    CTCCAGGTTTCCGGTTCCATCCAACCGCAAGAGGAGCTTCTTCACT  +1bp
Line1-4    CTCCAGGTTTCCGGTTCCATCCAACCGAAAGAGGAGCTTCTTCACT  +1bp
Line1-5    CTCCAGGTTTCCGGTTCCATCCAACCGCAAGAGGAGCTTCTTCACT  +1bp
Line1-6    CTCCAGGTTTCCGGTTCCATCCAACCGGAAGAGGAGCTTCTTCACT  +1bp
Line1-7    CTCCAGGTTTCCGGTTCCATCCAACCGTAAGAGGAGCTTCTTCACT  +1bp
Line1-8    CTCCAGGTTTCCGGTTCCATCCAACCGTAAGAGGAGCTTCTTCACT  +1bp
Line1-9    CTCCAGGTTTCCGGTTCCATCCAACCGAAAGAGGAGCTTCTTCACT  +1bp
Line1-10   CTCCAGGTTTCCGGTTCCATCCAACCGGAAGAGGAGCTTCTTCACT  +1bp
```

```
              PyNSC-gRNA01   pLR94      (10/10)  100%
WT         CTCCAGGTTTCCGGTTCCATCCAACCGAAGAGGAGCTTCTTCACT
Line2-1    CTCCAGGTTTCCGGTTCCATCCAACCGAAAGAGGAGCTTCTTCACT  +1bp
Line2-2    CTCCAGGTTTCCGGTTCCATCCAACC-AAGAGGAGCTTCTTCACT   -1bp
Line2-3    CTCCAGGTTTCCGGTTCCATCCAACCGAAAGAGGAGCTTCTTCACT  +1bp
Line2-4    CTCCAGGTTTCCGGTTCCATCCAACCGAAAGAGGAGCTTCTTCACT  +1bp
Line2-5    CTCCAGGTTTCCGGTTCCATCAA----AAGAGGAGCTTCTTCACT   -6bp,+2bp
Line2-6    CTCCAGGTTTCCGGTTCCATCCAAC--AAGAGGAGCTTCTTCACT   -2bp
Line2-7    CTCCAGGTTTCCGGTTCCATCCAACC-AAGAGGAGCTTCTTCACT   -1bp
Line2-8    CTCCAGGTTTCCGGTTCCATCCAACCGAAAGAGGAGCTTCTTCACT  +1bp
Line2-9    CTCCAGGTTTCCGGTTCCATCCAACCGAAAGAGGAGCTTCTTCACT  +1bp
Line2-10   CTCCAGGTTTCCGGTTCCATCCAACCGA---GGAGCTTCTTCACT   -3bp
```

```
              PyNSC-gRNA01   pLR94      (10/10)  100%
WT         CTCCAGGTTTCCGGTTCCATCCAACCGAAGAGGAGCTTCTTCACT
Line3-1    CTCCAGGTTTCCGGTTCCATCCAACCGAAAGAGGAGCTTCTTCACT  +1bp
Line3-2    CTCCAGGTTTCCGGTTCCATCCAACCGAAAGAGGAGCTTCTTCACT  +1bp
Line3-3    CTCCAGGTTTCCGGTTCCATCCAACCGAAAGAGGAGCTTCTTCACT  +1bp
Line3-4    CTCCAGGTTTCCGGTTCCATCCAACCGTAAGAGGAGCTTCTTCACT  +1bp
Line3-5    CTCCAGGTTTCCGGTTCCATCCAACCGGAAGAGGAGCTTCTTCACT  +1bp
Line3-6    CTCCAGGTTTCCGGTTCCATCCAACCGTAAGAGGAGCTTCTTCACT  +1bp
Line3-7    CTCCAGGTTTCCGGTTCCATCCAACCGTAAGAGGAGCTTCTTCACT  +1bp
Line3-8    CTCCAGGTTTCCGGTTCCATCCAACCGTAAGAGGAGCTTCTTCACT  +1bp
Line3-9    CTCCAGGTTTCCGGTTCCATCCAACCGAAAGAGGAGCTTCTTCACT  +1bp
Line3-10   CTCCAGGTTTCCGGTTCCATCCAACCGAAAGAGGAGCTTCTTCACT  +1bp
```

```
              PyMYB169-gRNA03   pLR92      (8/10)  80%
WT         AGTTGCAGGCTGAGGTGGACCAATTATCTGAGGCCAGACTTGAAG
Line1-1    AGTTGCAGGCTGAGGTGGACCAATTATTCTGAGGCCAGACTTGAAG  +1bp
Line1-2    AGTTGCAGGCTGAGGTGGACCAATTATC-----------------   -31bp
Line1-3    AGTTGCAGGCTGAGGTGGACCA-----CTGAGGCCAGACTTGAAG   -5bp
Line1-4    AGTTGCAGGCTGAGGTGGACCAATTATCTGAGGCCAGACTTGAAG
Line1-5    AGTTGCAGGCTGAGGTGGACCAATTATTCTGAGGCCAGACTTGAAG  +1bp
Line1-6    AGTTGCAGGCTGAGGTGGACCAATTATTCTGAGGCCAGACTTGAAG  +1bp
Line1-7    AGTTGCAGGCTGAGGTGGA-------TCTGAGGCCAGACTTGAAG   -7bp
Line1-8    AGTTGCAGGCTGAGGTGGACCAATTATTCTGAGGCCAGACTTGAAG  +1bp
Line1-9    AGTTGCAGGCTGAGGTGGACCAATTA-CTGAGGCCAGACTTGAAG   -1bp
Line1-10   AGTTGCAGGCTGAGGTGGACCAATTATCTGAGGCCAGACTTGAAG
```

```
              PyMYB169-gRNA03   pLR92      (10/10)  100%
WT         AGTTGCAGGCTGAGGTGGACCAATTATCTGAGGCCAGACTTGAAG
Line2-1    AGTTGCAGGCTGAGGTGGACC------CTGAGGCCAGACTTGAAG   -6bp
Line2-2    AGTTGCAGGCTGAGGTGGACCAATTATTCTGAGGCCAGACTTGAAG  +1bp
Line2-3    AGTTGCAGGCTGAGGTGGACCAATTATTCTGAGGCCAGACTTGAAG  +1bp
Line2-4    AGTTGCAGGCTGAGGTGGACCAATTATCCTGAGGCCAGACTTGAAG  +1bp
Line2-5    AGTTGCAGGCTGAGGTGGACCAAT---CTGAGGCCAGACTTGAAG   -3bp
Line2-6    AGTTGCAGGCTGAGGTGGACCAATTATTCTGAGGCCAGACTTGAAG  +1bp
Line2-7    AGTTGCAGGCTGAGGTGGACC------CTGAGGCCAGACTTGAAG   -6bp
Line2-8    AGTTGCAGGCTGAG------------------GCCAGACTTGAAG   -18bp
Line2-9    AGTTGCAGGCTGAGGTGGACCAA----CTGAGGCCAGACTTGAAG   -4bp
Line2-10   AGTTGCAGGCTGAGGTGGACCAATTAT------------------   -39bp
```

```
              PyMYB169-gRNA03   pLR92      (10/10)  100%
WT         AGTTGCAGGCTGAGGTGGACCAATTATCTGAGGCCAGACTTGAAG
Line3-1    AGTTGCAGGCTGAGGTGGACCAAT---CTGAGGCCAGACTTGAAG   -3bp
Line3-2    AGTTGCAGGCTGAGGTGGACCAATTA-----GGCCAGACTTGAAG   -5bp
Line3-3    AGTTGCAGGCTGAGGTGGACCA----TCTGAGGCCAGACTTGAAG   -4bp
Line3-4    AGTTGCAGGCTGAGGTGGACCA----TCTGAGGCCAGACTTGAAG   -4bp
Line3-5    AGTTGCAGGCTGAGGTGGACCAATTA-----GGCCAGACTTGAAG   -5bp
Line3-6    AGTTGCAGGCTGAGGTGGACCAATTA-----GGCCAGACTTGAAG   -5bp
Line3-7    AGTTGCAGGCTGAGGTGGACCAATTA-----GGCCAGACTTGAAG   -5bp
Line3-8    AGTTGCAGGCTGAGGTGGACCAAT---CTGAGGCCAGACTTGAAG   -3bp
Line3-9    AGTTGCAGGCTGAG------------------GCCAGACTTGAAG   -18bp
Line3-10   AGTTGCAGGCTGAG------------------GCCAGACTTGAAG   -18bp
```

```
              PyNSC-gRNA03   pLR94      (0/10)  0%
REF        CGGAACTCGAACCAATCGGGCAACCACCGCTGGGTTTTGGAAGGC
WT         CGGAACTCGAACCAATCGGGCAACCACtGCTGGGTTTTGGAAGGC
Line1-1    CGGAACTCGAACCAATCGGGCAACCACtGCTGGGTTTTGGAAGGC
Line1-2    CGGAACTCGAACCAATCGGGCAACCACtGCTGGGTTTTGGAAGGC
Line1-3    CGGAACTCGAACCAATCGGGCAACCACtGCTGGGTTTTGGAAGGC
Line1-4    CGGAACTCGAACCAATCGGGCAACCACtGCTGGGTTTTGGAAGGC
Line1-5    CGGAACTCGAACCAATCGGGCAACCACtGCTGGGTTTTGGAAGGC
Line1-6    CGGAACTCGAACCAATCGGGCAACCACtGCTGGGTTTTGGAAGGC
Line1-7    CGGAACTCGAACCAATCGGGCAACCACtGCTGGGTTTTGGAAGGC
Line1-8    CGGAACTCGAACCAATCGGGCAACCACtGCTGGGTTTTGGAAGGC
Line1-9    CGGAACTCGAACCAATCGGGCAACCACtGCTGGGTTTTGGAAGGC
Line1-10   CGGAACTCGAACCAATCGGGCAACCACtGCTGGGTTTTGGAAGGC
```

```
              PyNSC-gRNA03   pLR94      (0/10)  0%
REF        CGGAACTCGAACCAATCGGGCAACCACCGCTGGGTTTTGGAAGGC
WT         CGGAACTCGAACCAATCGGGCAACCACtGCTGGGTTTTGGAAGGC
Line2-1    CGGAACTCGAACCAATCGGGCAACCACtGCTGGGTTTTGGAAGGC
Line2-2    CGGAACTCGAACCAATCGGGCAACCACtGCTGGGTTTTGGAAGGC
Line2-3    CGGAACTCGAACCAATCGGGCAACCACtGCTGGGTTTTGGAAGGC
Line2-4    CGGAACTCGAACCAATCGGGCAACCACtGCTGGGTTTTGGAAGGC
Line2-5    CGGAACTCGAACCAATCGGGCAACCACtGCTGGGTTTTGGAAGGC
Line2-6    CGGAACTCGAACCAATCGGGCAACCACtGCTGGGTTTTGGAAGGC
Line2-7    CGGAACTCGAACCAATCGGGCAACCACtGCTGGGTTTTGGAAGGC
Line2-8    CGGAACTCGAACCAATCGGGCAACCACtGCTGGGTTTTGGAAGGC
Line2-9    CGGAACTCGAACCAATCGGGCAACCACtGCTGGGTTTTGGAAGGC
Line2-10   CGGAACTCGAACCAATCGGGCAACCACtGCTGGGTTTTGGAAGGC
```

```
              PyNSC-gRNA03   pLR94      (0/10)  0%
REF        CGGAACTCGAACCAATCGGGCAACCACCGCTGGGTTTTGGAAGGC
WT         CGGAACTCGAACCAATCGGGCAACCACtGCTGGGTTTTGGAAGGC
Line3-1    CGGAACTCGAACCAATCGGGCAACCACtGCTGGGTTTTGGAAGGC
Line3-2    CGGAACTCGAACCAATCGGGCAACCACtGCTGGGTTTTGGAAGGC
Line3-3    CGGAACTCGAACCAATCGGGCAACCACtGCTGGGTTTTGGAAGGC
Line3-4    CGGAACTCGAACCAATCGGGCAACCACtGCTGGGTTTTGGAAGGC
Line3-5    CGGAACTCGAACCAATCGGGCAACCACtGCTGGGTTTTGGAAGGC
Line3-6    CGGAACTCGAACCAATCGGGCAACCACtGCTGGGTTTTGGAAGGC
Line3-7    CGGAACTCGAACCAATCGGGCAACCACtGCTGGGTTTTGGAAGGC
Line3-8    CGGAACTCGAACCAATCGGGCAACCACtGCTGGGTTTTGGAAGGC
Line3-9    CGGAACTCGAACCAATCGGGCAACCACtGCTGGGTTTTGGAAGGC
Line3-10   CGGAACTCGAACCAATCGGGCAACCACtGCTGGGTTTTGGAAGGC
```

Figure S17. Guide RNA design for CRISPRa-based transcriptional activation. A, Schematics of the sgRNA positions and sequences of *PybZIPa, PyMYB114, PyMYB10, PybHLH3, PyDFR, PyANS* and *PyUFGT* for transcriptional activation. 20 nt protospacers with a NGG PAM are designed for dCas9. B, Vector construction of CRISPRa system for transcriptional activation of anthocyanin pathway genes.

A

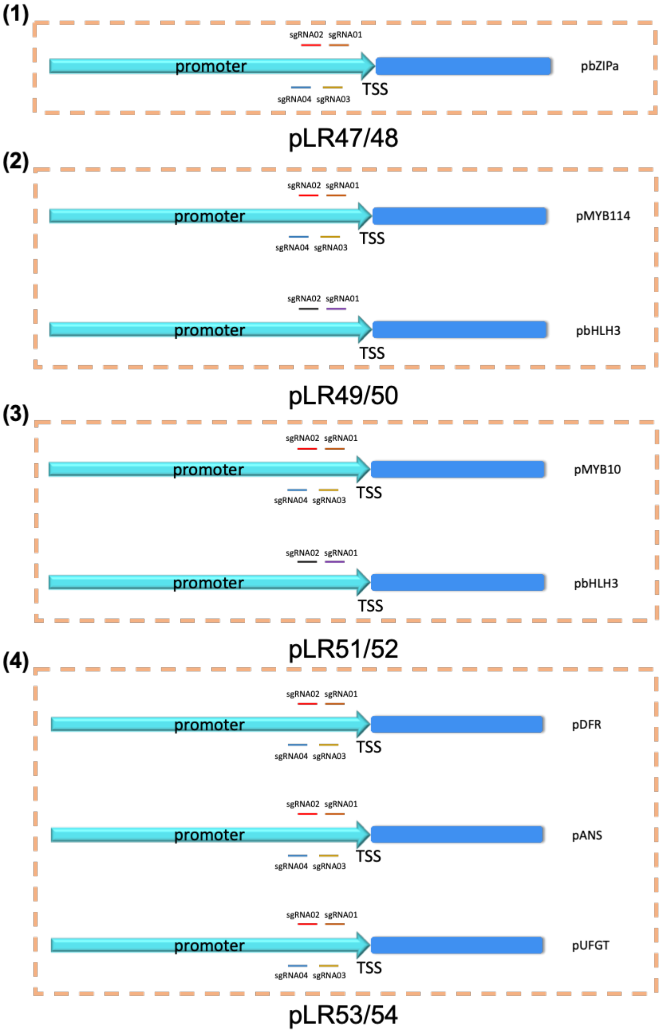

dCas9 [sgRNA: 20nt + PAM: NGG]

Target genes	sgRNAs	Target sequences	PAM
pbZIPa	sgRNA01	CTTCCTTTTACTCACAACCC	TGG
	sgRNA02	TGAAATGATACACAATATTA	AGG
	sgRNA03	TTGCCAGGGTTGTGAGTAAA	AGG
	sgRNA04	CTAGGAAGAAGAGTGTCGTG	CGG
pMYB114	sgRNA01	AGAGCAGTCATGCACGTCGG	TGG
	sgRNA02	GAAATACAACCTATCGTCTC	TGG
	sgRNA03	TGGAGAGCAGTCATGCACGT	CGG
	sgRNA04	AAAACGAATAGGTGTCAATT	GGG
pMYB10	sgRNA01	GAGTCTATGAAGTGGGTAGC	AGG
	sgRNA02	TATCCATAGCGTTAACGTCA	TGG
	sgRNA03	TCTTTCTGGAGTCTATGAAG	TGG
	sgRNA04	GTTAACGCTATGGATACCAT	CGG
pbHLH3	sgRNA01	TTATTTCAGAGGGAGAGTTA	GGG
	sgRNA02	GTGAGAAGGCAGCAGAAAGG	AGG
pDFR	sgRNA01	CAGAGAAAGCGGGCGTGAGC	GGG
	sgRNA02	CGGTTGACGCACGAGCGTGG	TGG
	sgRNA03	CTGTTTTATCTCAGCTTCCG	GGG
	sgRNA04	TTCTCCCTGATCTAATGGAG	AGG
pANS	sgRNA01	TGCAGGGCCTGCATTGTTAT	CGG
	sgRNA02	AGCGTGACCAAATGAGTAGT	TGG
	sgRNA03	TGATGAAGCAATGGTTTAAG	GGG
	sgRNA04	CGCTCACTTACTCTTGCTAG	TGG
pUFGT	sgRNA01	AATCCGGTGGCCCTCTGGG	GGG
	sgRNA02	TAGTAAAATTGGGAGTGCCG	TGG
	sgRNA03	TAGTAATCCGGTGGCCCTC	TGG
	sgRNA04	TGCAAATCAAGTAGTCTTGG	TGG

B

Vectors	Target genes	Target genes	Target genes	dCas9
pLR47	pbZIPa-gRNA01+gRNA02			AtUBQ10-dCas9
pLR48	pbZIPa-gRNA03+gRNA04			AtUBQ10-dCas9
pLR49	pMYB114-gRNA01+gRNA02	pbHLH3-gRNA01+gRNA02		AtUBQ10-dCas9
pLR50	pMYB114-gRNA03+gRNA04	pbHLH3-gRNA01+gRNA02		AtUBQ10-dCas9
pLR51	pMYB10-gRNA01+gRNA02	pbHLH3-gRNA01+gRNA02		AtUBQ10-dCas9
pLR52	pMYB10-gRNA03+gRNA04	pbHLH3-gRNA01+gRNA02		AtUBQ10-dCas9
pLR53	pDFR-gRNA01+gRNA02	pANS-gRNA01+gRNA02	pUFGT-gRNA01+gRNA02	AtUBQ10-dCas9
pLR54	pDFR-gRNA03+gRNA04	pANS-gRNA03+gRNA04	pUFGT-gRNA03+gRNA04	AtUBQ10-dCas9

Figure S18. PCR identification of the regenerated calli (pLR47-54) using vector-specific primers. M, DNA marker. Plasmid, positive control. WT, wide type. H_2O, negative control.

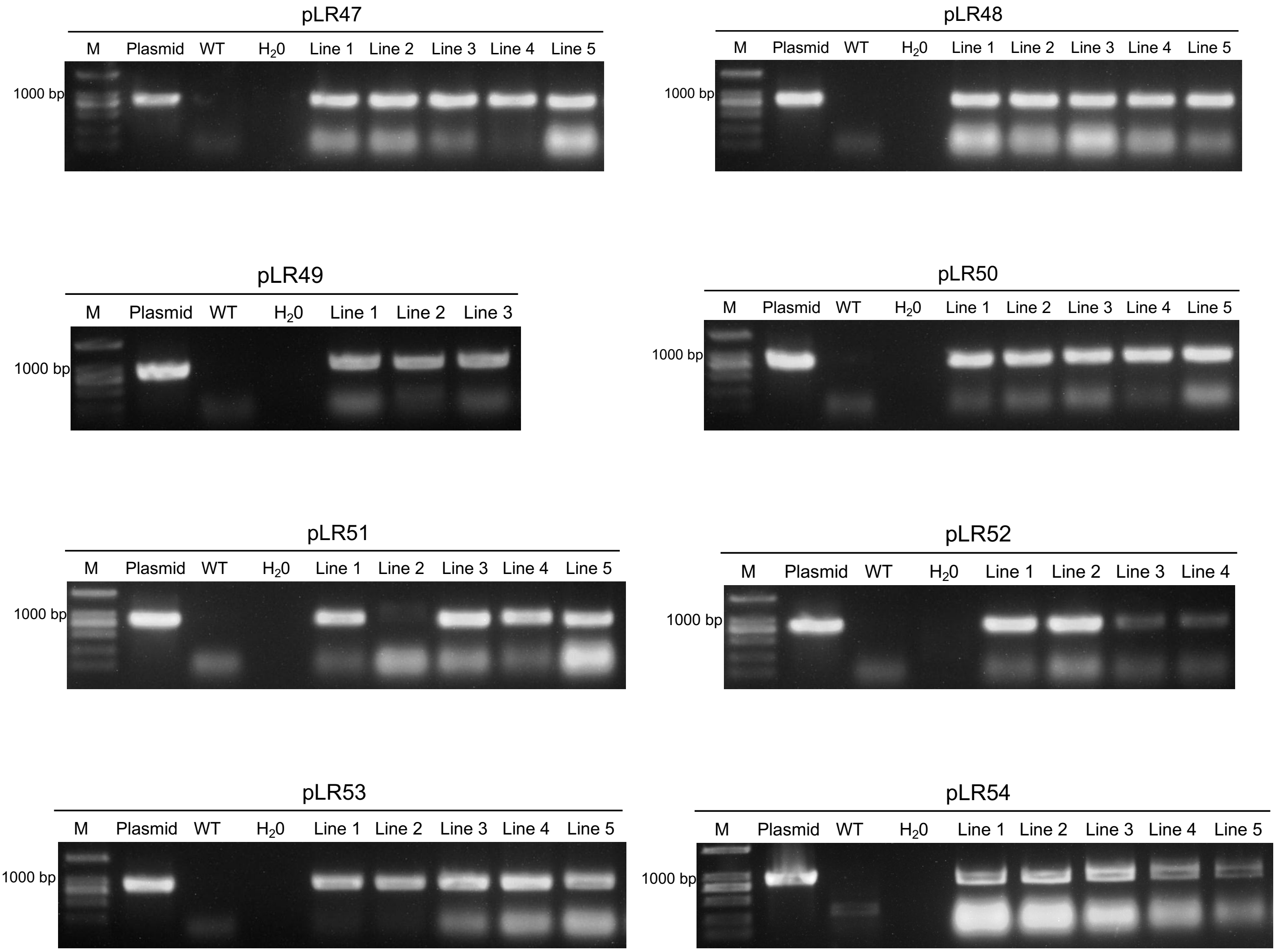

Figure S19. Sanger sequencing analysis of putative off-target sites for CRISPR/Cas9. The number represent mutated clones / sequenced clones. Dash indicates 1 bp deletion. Green DNA bases indicate insertion. The PAM sequence is highlighted in red, and the target sequence is highlighted in blue. WT, wild-type sequence.

PyPDS-gRNA02-mismatch 3 pLR04 (0/5) 0%

```
WT       GTTGATTAAAAGAAATTAACAGCACTCTCATGGAAAGGTAAAATT
Line1-1  GTTGATTAAAAGAAATTAACAGCACTCTCATGGAAAGGTAAAATT
Line1-2  GTTGATTAAAAGAAATTAACAGCACTCTCATGGAAAGGTAAAATT
Line1-3  GTTGATTAAAAGAAATTAACAGCACTCTCATGGAAAGGTAAAATT
Line1-4  GTTGATTAAAAGAAATTAACAGCACTCTCATGGAAAGGTAAAATT
Line1-5  GTTGATTAAAAGAAATTAACAGCACTCTCATGGAAAGGTAAAATT
```

PyTFL1.1-gRNA03-mismatch 3 pLR14 (0/5) 0%

```
WT       GCTGAGCTACGAGATGCCAAGGCCCAACATTGGCATCCACAGGTT
Line1-1  GCTGAGCTACGAGATGCCAAGGCCCAACATTGGCATCCACAGGTT
Line1-2  GCTGAGCTACGAGATGCCAAGGCCCAACATTGGCATCCACAGGTT
Line1-3  GCTGAGCTACGAGATGCCAAGGCCCAACATTGGCATCCACAGGTT
Line1-4  GCTGAGCTACGAGATGCCAAGGCCCAACATTGGCATCCACAGGTT
Line1-5  GCTGAGCTACGAGATGCCAAGGCCCAACATTGGCATCCACAGGTT
```

PyMYB114-gRNA03-mismatch 2 pLR91 (3/5) 60%

```
WT       AGAAAGCTGGAAAGATCGGAGATTTCACAGTGGAGATAGAGAGAG
Line1-1  AGAAAGCTGGAAAGATCGGAGATTTCAACAGTGGAGATAGAGAGAG   +1bp
Line1-2  AGAAAGCTGGAAAGATCGGAGATTTCA-AGTGGAGATAGAGAGAG    -1bp
Line1-3  AGAAAGCTGGAAAGATCGGAGATTTCACAGTGGAGATAGAGAGAG
Line1-4  AGAAAGCTGGAAAGATCGGAGATTTCA--GTGGAGATAGAGAGAG    -2bp
Line1-5  AGAAAGCTGGAAAGATCGGAGATTTCACAGTGGAGATAGAGAGAG
```

PyMYB114-gRNA03-mismatch 3-1 pLR91 (0/5) 0%

```
WT       AGCCGAATCTCAAAAGCGGACATTTTACAGAGGATGAAATAGATC
Line1-1  AGCCGAATCTCAAAAGCGGACATTTTACAGAGGATGAAATAGATC
Line1-2  AGCCGAATCTCAAAAGCGGACATTTTACAGAGGATGAAATAGATC
Line1-3  AGCCGAATCTCAAAAGCGGACATTTTACAGAGGATGAAATAGATC
Line1-4  AGCCGAATCTCAAAAGCGGACATTTTACAGAGGATGAAATAGATC
Line1-5  AGCCGAATCTCAAAAGCGGACATTTTACAGAGGATGAAATAGATC
```

PyMYB114-gRNA03-mismatch 3-2 pLR91 (0/5) 0%

```
WT       AGCCAAATATCAAGAGAGGAGACTTTACAGAGGATGAAGTAGATC
Line1-1  AGCCAAATATCAAGAGAGGAGACTTTACAGAGGATGAAGTAGATC
Line1-2  AGCCAAATATCAAGAGAGGAGACTTTACAGAGGATGAAGTAGATC
Line1-3  AGCCAAATATCAAGAGAGGAGACTTTACAGAGGATGAAGTAGATC
Line1-4  AGCCAAATATCAAGAGAGGAGACTTTACAGAGGATGAAGTAGATC
Line1-5  AGCCAAATATCAAGAGAGGAGACTTTACAGAGGATGAAGTAGATC
```

PyGID1-gRNA05-mismatch 2 pLR10 (2/5) 40%

```
WT       TCATATCTATCTAGCTGGCGATAGCTCTGGCGGGAACATTGTACA
Line1-1  TCATATCTATCTAGCTGGCGATAGCTCTGGCGGGAACATTGTACA
Line1-2  TCATATCTATCTAGCTGGCGATAGCTCTGGCGGGAACATTGTACA
Line1-3  TCATATCTATCTAGCTGGCGATAGCTCTGGGCGGGAACATTGTACA   +1bp
Line1-4  TCATATCTATCTAGCTGGCGATAGCTCTGGCGGGAACATTGTACA
Line1-5  TCATATCTATCTAGCTGGCGATAGCTCTGGGTGGGAACATTGTACA   -1bp,+2bp
```

PyMYB10-gRNA03-mismatch 2 pLR91 (0/5) 0%

```
WT       GTCATCACAAACGTCGTCGTCGACAAACAATGGAAATGATTGGTG
Line1-1  GTCATCACAAACGTCGTCGTCGACAAACAATGGAAATGATTGGTG
Line1-2  GTCATCACAAACGTCGTCGTCGACAAACAATGGAAATGATTGGTG
Line1-3  GTCATCACAAACGTCGTCGTCGACAAACAATGGAAATGATTGGTG
Line1-4  GTCATCACAAACGTCGTCGTCGACAAACAATGGAAATGATTGGTG
Line1-5  GTCATCACAAACGTCGTCGTCGACAAACAATGGAAATGATTGGTG
```

PyMYB10-gRNA03-mismatch 3-1 pLR91 (0/5) 0%

```
WT       GCCATCACAAACGTTGTCATCGACAAAGAATGGAAATGATTGGTG
Line1-1  GCCATCACAAACGTTGTCATCGACAAAGAATGGAAATGATTGGTG
Line1-2  GCCATCACAAACGTTGTCATCGACAAAGAATGGAAATGATTGGTG
Line1-3  GCCATCACAAACGTTGTCATCGACAAAGAATGGAAATGATTGGTG
Line1-4  GCCATCACAAACGTTGTCATCGACAAAGAATGGAAATGATTGGTG
Line1-5  GCCATCACAAACGTTGTCATCGACAAAGAATGGAAATGATTGGTG
```

PyMYB10-gRNA03-mismatch 3-2 pLR91 (0/5) 0%

```
WT       GTTATCTGTTTCTTCATCGTCAACAAAGAATGGGAACTGCAAAAC
Line1-1  GTTATCTGTTTCTTCATCGTCAACAAAGAATGGGAACTGCAAAAC
Line1-2  GTTATCTGTTTCTTCATCGTCAACAAAGAATGGGAACTGCAAAAC
Line1-3  GTTATCTGTTTCTTCATCGTCAACAAAGAATGGGAACTGCAAAAC
Line1-4  GTTATCTGTTTCTTCATCGTCAACAAAGAATGGGAACTGCAAAAC
Line1-5  GTTATCTGTTTCTTCATCGTCAACAAAGAATGGGAACTGCAAAAC
```

PyMYB10-gRNA03-mismatch 3-3 pLR91 (0/5) 0%

```
WT       GTCATCACAAACGTCGTCGTTGACAAACAATGGAAATGATTGGTG
Line1-1  GTCATCACAAACGTCGTCGTTGACAAACAATGGAAATGATTGGTG
Line1-2  GTCATCACAAACGTCGTCGTTGACAAACAATGGAAATGATTGGTG
Line1-3  GTCATCACAAACGTCGTCGTTGACAAACAATGGAAATGATTGGTG
Line1-4  GTCATCACAAACGTCGTCGTTGACAAACAATGGAAATGATTGGTG
Line1-5  GTCATCACAAACGTCGTCGTTGACAAACAATGGAAATGATTGGTG
```
